# Convergent synthesis of the tetrasaccharide repeating unit of the *O*-antigen of *Shigella boydii* type 9

**DOI:** 10.3762/bjoc.7.137

**Published:** 2011-08-29

**Authors:** Abhishek Santra, Anup Kumar Misra

**Affiliations:** 1 Bose Institute, Division of Molecular Medicine, P-1/12, C.I.T. Scheme VII-M, Kolkata-700054, India; Fax: 91-33-2355 3886

**Keywords:** diarrhea, glycosylation, *O*-antigen, oligosaccharide, *Shigella boydii*

## Abstract

A convenient synthesis of the tetrasaccharide repeating unit of the *O*-antigen of *Shigella boydii* type 9 has been achieved in excellent yield using a [2 + 2] block glycosylation strategy. TEMPO-mediated selective oxidation of the primary alcohol of the tetrasaccharide derivative **8** to the carboxylic group followed by deprotection of the functional groups furnished target tetrasaccharide **1** as its 4-methoxyphenyl glycoside in high yield.

## Introduction

**Figure 1:**
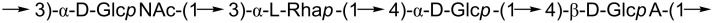
Structure of the tetrasaccharide repeating unit of the *O*-antigen of *Shigella boydii* type 9.

Diarrhoeal disease is a common cause of death in the tropical countries and it is the second mostly causing infant deaths worldwide. *Shigella* is one of the well-studied human pathogens that cause diarrhoeal disease and dysentery (e.g., shigellosis). Among several types of *Shigella* species, *Shigella dysenteriae* is the most virulent pathogen causing devastating health problems in developing countries [1–3]. *Shigella* strains are classified into four species: *Shigella boydii*, *Shigella dysenteriae*, *Shigella flexneri* and *Shigella sonnei* [4]. Sometimes, these species are also termed as *Shigella* subgroups A, B, C, and D. Based on the *O*-antigens, the *Shigella* species are divided into multiple serotypes [5]. In general, *O*-antigens of *Shigella* species are acidic in nature because of the presence of acidic constituents (e.g., uronic acid, pseudaminic acid etc. or lactic acid, pyruvic acid etc.) in their structures [6–7]. Recently, L’vov et al. reported the structure of the *O*-antigen of *Shigella boydii* type 9, which is a tetrasaccharide repeating unit containing a D-glucuronic acid moiety (Figure 1) [8].

**Figure 2:**
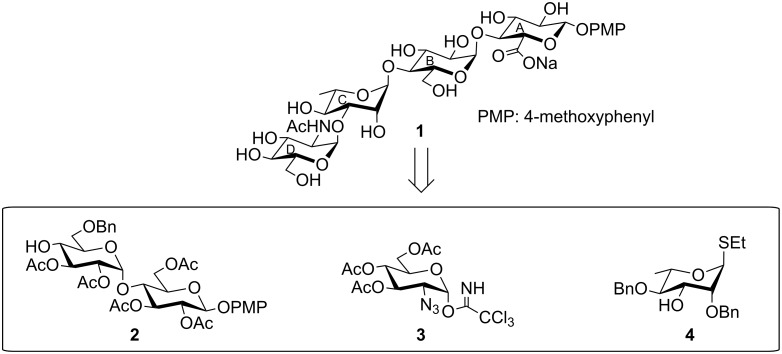
Structure of the synthesized tetrasaccharide and its intermediates.

Development of effective therapeutics to control the infections of drug-resistant bacterial strains is the thrust area in the medicinal chemistry. Like other bacterial infections, emergence of the drug resistant *Shigella* infections requires development of the newer therapeutics than the earlier used anti-shigellosis agents [9–10]. Because of the high antigenic nature of the *O*-antigens, antibodies against the *O*-specific polysaccharide of a particular *Shigella* strain have the potential to control *Shigella* infections [11–14]. A number of reports have been cited earlier to develop glycoconjugate based therapy to control *Shigella* infections [15–19]. In order to develop a glycoconjugate based therapeutic agent from the tetrasaccharide repeating unit of the *O*-antigen of *Shigella boydii* type 9, it is essential to perform several immunochemical studies with the glycoconjugates derived from this tetrasaccharide repeating unit. The large quantity of the tetrasaccharide that is required for this purpose cannot be accessible from a natural source. Therefore, chemical synthesis is the only option for achieving this in large quantity. As a first step towards the preparation of glycoconjugates, we report herein a convergent chemical synthesis of the tetrasaccharide as its 4-methoxyphenyl glycoside **1** corresponding to the *O*-antigen of *Shigella boydii* type 9 using a [2 + 2] block glycosylation strategy (Figure 2).

## Results and Discussion

The convergent synthesis of the target tetrasaccharide **1** as its 4-methoxyphenyl glycoside has been achieved applying a number of recently developed elegant reaction methodologies. A number of notable features are present in the synthetic strategy, which are (a) convergent [2 + 2] block glycosylation; (b) application of recently developed environmentally benign reaction conditions for protecting group manipulations and glycosylations such as, (i) *O*-acetylation using sulfamic acid [20], (ii) regioselective ring opening of the benzylidene acetal using a combination of triethylsilane and iodine [21], (iii) direct one-pot conversion of *O*-acetyl group to *O*-benzyl group [22], (iv) activation of glycosyl trichloroacetimidate and thioglycoside donors by perchloric acid supported on silica (HClO_4_–SiO_2_) [23–26], and late stage TEMPO mediated selective oxidation [27–29] of the primary hydroxy group to the carboxylic group under phase transfer conditions without affecting the secondary hydroxy groups; (c) use of 4-methoxyphenyl (PMP) group as anomeric protecting group [30–31], which can be easily removed under oxidative conditions for the preparation of glycoconjugate derivatives. The synthesis of the target tetrasaccharide **1** was achieved by the stereoselective coupling of a D-maltose derived disaccharide derivative **2** and a disaccharide thioglycoside derivative **7** followed by functional group manipulations of the resulting tetrasaccharide derivative **8**. For this purpose, suitably functionalized reaction intermediates **2**, **3**, **4** and **7** were prepared from commercially available reducing mono- and disaccharides utilizing a series of reaction methodologies reported earlier.

**Scheme 1:**
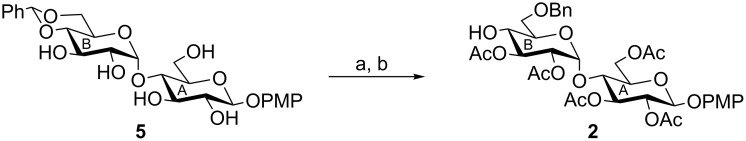
Reagents: (a) acetic anhydride, sulfamic acid, 60 °C, 30 min, 91%; (b) Et_3_SiH, I_2_, 0–5 °C, 30 min, 82%.

**Scheme 2:**
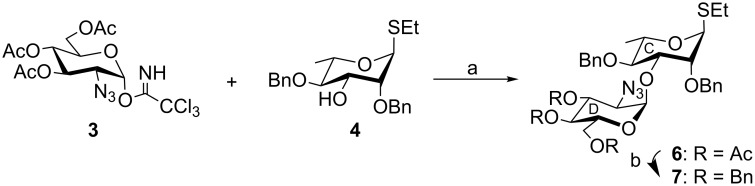
Reagents: (a) HClO_4_–SiO_2_, CH_2_Cl_2_, −15 °C, 1 h, 81%; (b) benzyl bromide, NaOH, *n*-Bu_4_NBr, THF, rt, 2 h, 91%.

**Scheme 3:**
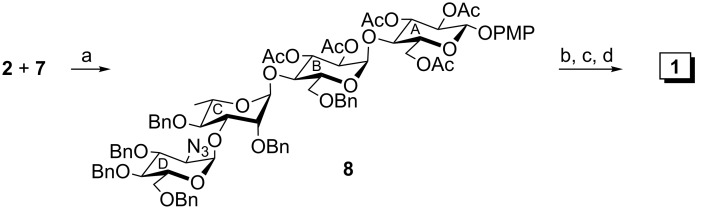
Reagents: (a) *N*-iodosuccinimide, HClO_4_–SiO_2_, −10 °C, 1 h, 82%; (b) 0.1 M CH_3_ONa, CH_3_OH, rt, 3 h; (c) (i) NaBr, TBAB, TEMPO, CH_2_Cl_2_, H_2_O, NaOCl, NaHCO_3_, 5 °C, 2 h; (ii) *tert*-butanol, 2-methylbut-2-ene, NaClO_2_, NaH_2_PO_4_, rt, 3 h; (d) (i) H_2_, 20% Pd(OH)_2_/C, CH_3_OH–EtOAc, rt, 10 h; (ii) acetic anhydride, CH_3_OH, rt, 30 min, overall 64%.

4-Methoxyphenyl (4,6-*O*-benzylidene-α-D-glucopyranosyl)-(1→4)-β-D-glucopyranoside (**5**) [32] was subjected to a sequence of reactions involving acetylation using acetic anhydride in the presence of sulfamic acid [20] followed by regioselective reductive ring opening of the benzylidene acetal using a combination of triethylsilane and iodine [21] to furnish 4-methoxyphenyl (2,3-di-*O*-acetyl-6-*O*-benzyl-α-D-glucopyranosyl)-(1→4)-2,3,6-tri-*O*-acetyl-β-D-glucopyranoside (**2**) in 82% yield (Scheme 1). 3,4,6-Tri-*O*-acetyl-2-azido-2-deoxy-α-D-glucopyranosyl trichloroacetimidate (**3**) [33] was allowed to couple stereoselectively with ethyl 2,4-di-*O*-benzyl-1-thio-α-L-rhamnopyranoside (**4**) [34] under Schmidt’s reaction conditions [35] using perchloric acid supported on silica (HClO_4_–SiO_2_) [23–24] as glycosylation activator to give ethyl (3,4,6-tri-*O*-acetyl-2-azido-2-deoxy-α-D-glucopyranosyl)-(1→3)-2,4-di-*O*-benzyl-1-thio-α-L-rhamnopyranoside (**6**) in 81% yield. Stereoselective formation of compound **6** was confirmed from its spectral analysis (presence of signals at δ 5.35 (br s, H-1_C_), 4.95 (d, *J* = 3.6 Hz, H-1_D_) in the ^1^H NMR and signals at δ 92.8 (C-1_D_), 81.0 (C-1_C_) in the ^13^C NMR spectrum). Compound **6** was transformed into disaccharide thioglycoside donor **7** in 91% yield under a one-pot deacetylation–benzylation reaction condition [22] (Scheme 2). In this case, the thioethyl group acts as an orthogonal anomeric protecting group since it acts as a glycosyl acceptor in the case of compound **4** whereas compound **7** has been used as the glycosyl donor in the next step. Iodonium ion promoted stereoselective glycosylation of the disaccharide thioglycoside donor **7** with the disaccharide acceptor **2** in the presence of a combination of *N*-iodosuccinimide (NIS) and HClO_4_–SiO_2_ [25–26] furnished tetrasaccharide derivative **8** in 82% yield. Stereoselective formation of new α-glycosyl linkage in compound **8** was confirmed from the 1D and 2D NMR spectral analysis (presence of signals at δ 5.34 (d, *J* = 4.5 Hz, H-1_B_), 4.97 (d, *J* = 3.5 Hz, H-1_D_), 4.95 (d, *J* = 8.0 Hz, H-1_A_), 4.89 (d, *J* = 2.0 Hz, H-1_C_) in the ^1^H NMR and signals at δ 99.7 (C-1_A_), 98.8 (C-1_C_), 96.0 (C-1_B_), 93.9 (C-1_D_) in the ^13^C NMR spectrum). Because of the presence of the non-participating benzyloxy group in the C-2 position of the L-rhamnosyl moiety, involved in the glycosylation reaction, a minor amount of β-glycosyl linked product also formed (~6%) together with the desired product **8**, which was separated by the column chromatography. The presence of the α-linkages in compound **8** was further confirmed from the *J*_C1-H1_ values in the ^1^H coupled ^13^C NMR spectrum of compound **8**. Appearance of *J*_C1-H1_ 172.0 Hz, 171.6 Hz, 170.5 Hz and 162.0 Hz values in the anomeric region in the ^1^H coupled ^13^C spectrum unambiguously supported the presence of three α-linkages and one β-linkage [36–38] in compound **8**. Compound **8** was subjected to a reaction sequence involving (a) deacetylation using 0.1 M sodium methoxide in methanol; (b) TEMPO mediated selective oxidation [27–29] of the primary hydroxy group leaving secondary hydroxy groups unaffected in a phase transfer reaction condition and (c) removal of benzyl groups for cleavage of ethers and reduction of the azido group to an amine by hydrogenation over 20% Pd(OH)_2_/C followed by *N*-acetylation to furnish target tetrasaccharide **1** as its sodium salt and 4-methoxyphenyl glycoside in 64% yield (Scheme 3). Spectroscopic analysis of compound **1** confirmed its formation (presence of signals at δ 5.20 (d, *J* = 3.6 Hz, H-1_B_), 4.93 (br s, H-1_D_), 4.89 (br s, H-1_C_), 4.82 (d, *J* = 7.8 Hz, H-1_A_) in the ^1^H NMR and signals at δ 103.3 (C-1_A_), 102.8 (C-1_B_), 102.4 (C-1_C_), 96.7 (C-1_D_) in the ^13^C NMR spectrum).

## Conclusion

In conclusion, a convenient synthetic strategy has been developed for the synthesis of the tetrasaccharide repeating unit of the *O*-antigen of *Shigella boydii* type 9 as its 4-methoxyphenyl glycoside sodium salt using a [2 + 2] block synthetic strategy. Use of a block glycosylation strategy and a late-stage selective oxidation of the primary hydroxy group significantly reduced the number of protection–deprotection steps. A number of modified clean reaction methodologies have been applied for the preparation of intermediates. HClO_4_–SiO_2_ has been used as an effective acid catalyst to activate glycosyl trichloroacetimidate derivative and thioglycoside in combination with NIS avoiding the use of moisture sensitive protic acids. All intermediate steps were high yielding and glycosylation steps were stereoselective.

## Experimental

**General methods:** All reactions were monitored by thin layer chromatography over silica gel-coated TLC plates. The spots on TLC plates were visualized by warming ceric sulfate (2% Ce(SO_4_)_2_ in 2 N H_2_SO_4_)-sprayed plates on a hot plate. Silica gel 230–400 mesh was used for column chromatography. ^1^H and ^13^C NMR, DEPT 135, 2D COSY, HMQC and gated ^1^H coupled ^13^C NMR spectra were recorded on Bruker Avance DRX 500 and 600 MHz spectrometers using CDCl_3_ and CD_3_OD as solvents and TMS as internal standard unless otherwise stated. Chemical shifts are expressed in δ ppm. ESIMS were recorded on a Micromass Quattro mass spectrometer. Elementary analysis was carried out on Carlo Erba-1108 analyzer. Optical rotations were measured at 25 °C on a Jasco P-2000 polarimeter. Commercially available grades of organic solvents of adequate purity are used in all reactions. Silica supported perchloric acid (HClO_4_–SiO_2_) was prepared following the earlier report [39].

**4-Methoxyphenyl (2,3-di-*O*-acetyl-6-*O*-benzyl-α-D-glucopyranosyl)-(1→4)-2,3,6-tri-*O*-acetyl-β-D-glucopyranoside (2):** To a suspension of 4-methoxyphenyl (4,6-*O*-benzylidene-α-D-glucopyranosyl)-(1→4)-β-D-glucopyranoside (**5**, 3.0 g, 5.59 mmol) in acetic anhydride (6.0 mL, 63.47 mmol) sulfamic acid (100 mg, 1.0 mmol) was added and the reaction mixture was allowed to stir at 60 °C for 30 min. The solvents were removed under reduced pressure and the crude reaction mixture was passed through a short pad of silica gel using hexane–EtOAc (1:2) as eluant to give pure acetylated product (3.8 g, 91%). To a solution of the acetylated product (3.5 g, 4.69 mmol) in CH_3_CN (15 mL) triethylsilane (1.5 mL, 9.39 mmol) and iodine (250 mg, 0.98 mmol) were added at 0–5 °C and the reaction mixture was allowed to stir at the same temperature for 30 min. The reaction mixture was poured into water and extracted with CH_2_Cl_2_ (100 mL). The organic layer was successively washed with satd. NaHCO_3_ solution and water, dried (Na_2_SO_4_) and concentrated. The crude product was purified over silica gel using hexane–EtOAc (2:1) as eluant to give pure compound **2** (2.9 g, 82%). White solid; mp 60–62 °C; [α]_D_^25^ +43.2 (*c* 1.2, CHCl_3_); IR (KBr): 3479, 1751, 1509, 1371, 1233, 1047, 830, 754 cm^−1^; ^1^H NMR (500 MHz, CDCl_3_) δ 5.38 (d, *J* = 4.0 Hz, 1H, H-1_B_), 5.28 (t, *J* = 9.0 Hz, 1H, H-3_A_), 5.21 (t, *J* = 9.0 Hz, 1H, H-3_B_), 5.04 (t, *J* = 8.5 Hz, 1H, H-2_A_), 4.96 (d, *J* = 8.0 Hz, 1H, H-1_A_), 4.80 (dd, *J* = 9.0, 4.0 Hz, 1H, H-2_B_), 4.60–4.54 (m, 2H, PhC*H*_2_), 4.50 (d, *J* = 11.5 Hz, 1H, H-6_aA_), 4.22 (dd, *J* = 12.0 Hz, 5.0 Hz, 1H, H-6_bA_), 4.06 (t, *J* = 9.5 Hz, 1H, H-4_A_), 3.79–3.71 (m, 4H, H-4_B_, H-5_A_, H-5_B_, H-6_aB_), 3.75 (s, 3H, OC*H*_3_), 3.63–3.61 (m, 1H, H-6_bB_), 2.07, 2.03, 2.00, 1.99 (4 s, 15H, 5 COC*H*_3_); ^13^C NMR (125 MHz, CDCl_3_) δ 170.1, 170.6, 170.5, 170.2, 169.6 (5 *C*OCH_3_), 155.6–114.5 (Ar-C), 99.6 (C-1_A_), 95.7 (C-1_B_), 75.4 (C-3_A_), 73.7 (Ph*C*H_2_), 72.3 (C-4_A_), 72.1 (C-3_B_), 72.0 (C-2_A_), 71.9 (C-5_A_), 71.5 (C-5_B_), 70.0 (C-2_B_), 69.7 (C-4_B_), 68.8 (C-6_B_), 62.7 (C-6_A_), 55.6 (O*C*H_3_), 20.8, 20.7, 20.6, 20.5 (2 C) (5 CO*C*H_3_); ESIMS *m*/*z*: 771.2 [M + Na]^+^; anal. calcd for C_36_H_44_O_17_ (748.26): C, 57.75; H, 5.92; found: C, 57.54; H, 6.15.

**Ethyl (3,4,6-tri-*O*-acetyl-2-azido-2-deoxy-α-D-glucopyranosyl)-(1→3)-2,4-di-*O*-benzyl-1-thio-α-L-rhamnopyranoside (6):** A solution of compound **3** (2.4 g, 5.04 mmol) and compound **4** (1.5 g, 3.86 mmol) in anhydrous CH_2_Cl_2_ (10 mL) was cooled to −15 °C under argon. To the cooled reaction mixture HClO_4_–SiO_2_ (50.0 mg) was added and the reaction mixture was allowed to stir at the same temperature for 1 h. The reaction mixture was filtered through a bed of Celite^®^ and concentrated under reduced pressure. The crude product was purified over silica gel using hexane–EtOAc (3:1) as eluant to furnish pure compound **6** (2.2 g, 81%). Yellow oil; [α]_D_^25^ +63.3 (*c* 1.2, CHCl_3_); IR (neat): 2930, 2110, 1750, 1455, 1368, 1233, 1094, 1039, 755, 699 cm^−1^; ^1^H NMR (600 MHz, CDCl_3_) δ 7.43–7.26 (m, 10H, Ar-H), 5.55 (t, *J* = 10.2 Hz, 1H, H-3_D_), 5.35 (br s, 1H, H-1_C_), 4.98 (t, *J* = 10.2 Hz, 1H, H-4_D_), 4.95 (d, *J* = 3.6 Hz, 1H, H-1_D_), 4.91 (d, *J* = 11.4 Hz, 1H, PhC*H*_2_), 4.74 (d, *J* = 12.0 Hz, 1H, PhC*H*_2_), 4.70 (d, *J* = 11.4 Hz, 1H, PhC*H*_2_), 4.64 (d, *J* = 12.0 Hz, 1H, PhC*H*_2_), 4.15–4.13 (m, 1H, H-5_D_), 4.08–4.03 (m, 2H, H-5_C_, H-6_aD_), 4.00 (dd, *J* = 9.6, 3.0 Hz, 1H, H-3_C_), 3.93 (br s, 1H, H-2_C_), 3.88 (d, *J* = 12.0 Hz, 1H, H-6_bD_), 3.69 (t, *J* = 9.6 Hz, 1H, H-4_C_), 3.31 (dd, *J* = 10.8, 3.6 Hz, 1H, H-2_D_), 2.61–2.56 (m, 2H, SC*H*_2_CH_3_), 2.08, 2.05, 1.90 (3 s, 9H, 3 COC*H*_3_), 1.36 (d, *J* = 6.0 Hz, 3H, CC*H*_3_), 1.25 (t, *J* = 7.2 Hz, 3H, SCH_2_C*H*_3_); ^13^C NMR (150 MHz, CDCl_3_) δ 170.5, 169.8, 169.6 (3 *C*OCH_3_), 138.0–127.6 (Ar-C), 92.8 (C-1_D_), 81.0 (C-1_C_), 79.4 (C-3_C_), 75.5 (Ph*C*H_2_), 74.6 (C-3_C_), 74.2 (C-2_C_), 71.8 (Ph*C*H_2_), 70.4 (C-3_D_), 68.4 (C-5_C_), 68.1 (C-4_D_), 67.3 (C-5_D_), 61.5 (C-6_D_), 60.6 (C-2_D_), 25.4 (S*C*H_2_CH_3_), 20.7, 20.6, 20.5 (3 CO*C*H_3_), 17.7 (C*C*H_3_), 14.9 (SCH_2_*C*H_3_); ESIMS *m*/*z*: 724.2 [M + Na]^+^; anal. calcd for C_34_H_43_N_3_O_11_S (701.26): C, 58.19; H, 6.18; found: C, 58.0; H, 6.42.

**Ethyl (2-azido-3,4,6-tri-*O*-benzyl-2-deoxy-α-D-glucopyranosyl)-(1→3)-2,4-di-*O*-benzyl-1-thio-α-L-rhamnopyranoside (7):** To a solution of compound **6** (2.0 g, 2.85 mmol) in THF (10 mL) powdered NaOH (1.0 g, 25 mmol), benzyl bromide (2.1 mL, 17.6 mmol) and *n*-Bu_4_NBr (50 mg) were added and the reaction mixture was allowed to stir at rt for 2 h. The reaction mixture was poured into water and extracted with CH_2_Cl_2_ (100 mL). The organic layer was washed with water, dried (Na_2_SO_4_) and concentrated under reduced pressure. The crude product was purified over silica gel using hexane–EtOAc (6:1) as eluant to give pure compound **7** (2.2 g, 91%). Yellow oil; [α]_D_^25^ +12.0 (*c* 1.2, CHCl_3_); IR (neat): 3430, 3031, 2925, 2107, 1642, 1496, 1454, 1361, 1215, 1091, 1051, 1028, 751, 697 cm^−1^; ^1^H NMR (600 MHz, CDCl_3_) δ 7.45–7.07 (m, 25H, Ar-H), 5.32 (br s, 1H, H-1_C_), 4.93 (d, *J* = 3.6 Hz, 1H, H-1_D_), 4.85 (br s, 2H, PhC*H*_2_), 4.83 (d, *J* = 10.2 Hz, 1H, PhC*H*_2_), 4.77 (d, *J* = 10.8 Hz, 1H, PhC*H*_2_), 4.74 (d, *J* = 12.0 Hz, 1H, PhC*H*_2_), 4.69 (d, *J* = 12.0 Hz, 1H, PhC*H*_2_), 4.60 (d, *J* = 12.6 Hz, 1H, PhC*H*_2_), 4.55 (d, *J* = 10.2 Hz, 1H, PhC*H*_2_), 4.48 (d, *J* = 10.8 Hz, 1H, PhC*H*_2_), 4.35 (d, *J* = 12.6 Hz, 1H, PhC*H*_2_), 4.07 (t, *J* = 9.6 Hz, 1H, H-3_D_), 4.04–3.99 (m, 3H, H-3_C_, H-5_C_, H-5_D_), 3.93 (br s, 1H, H-2_C_), 3.79 (t, *J* = 9.6 Hz, 1H, H-4_C_), 3.66 (t, *J* = 9.6 Hz, 1H, H-4_D_), 3.60 (dd, *J* = 10.8, 2.4 Hz, 1H, H-6_aD_), 3.51 (dd, *J* = 10.8, 1.2 Hz, 1H, H-6_aD_), 3.40 (dd, *J* = 10.2, 3.6 Hz, 1H, H-2_D_), 2.60–2.50 (m, 2H, SC*H*_2_CH_3_), 1.35 (d, *J* = 6.0 Hz, 3H, CC*H*_3_), 1.22 (t, *J* = 7.8 Hz, 3H, SCH_2_C*H*_3_); ^13^C NMR (150 MHz, CDCl_3_) δ 137.7–127.5 (Ar-C), 93.2 (C-1_D_), 81.1 (C-1_C_), 80.2 (C-3_D_), 79.8 (C-4_D_), 78.1 (C-4_C_), 75.9 (Ph*C*H_2_), 74.8 (Ph*C*H_2_), 74.5 (C-3_C_), 74.4 (C-2_C_), 73.3 (Ph*C*H_2_), 71.8 (Ph*C*H_2_), 70.4 (C-5_C_), 68.3 (C-5_D_), 67.8 (C-6_D_), 63.0 (C-2_D_), 25.4 (S*C*H_2_CH_3_), 17.8 (C*C*H_3_), 14.9 (SCH_2_*C*H_3_); ESIMS *m*/*z*: 868.3 [M + Na]^+^; anal. calcd for C_49_H_55_N_3_O_8_S (845.37): C, 69.56; H, 6.55; found: C, 69.33; H, 6.80.

**4-Methoxyphenyl (2-azido-3,4,6-tri-*O*-benzyl-2-deoxy-α-D-glucopyranosyl)-(1→3)-(2,4-di-*O*-benzyl-α-L-rhamnopyranosyl)-(1→4)-(2,3-di-*O*-acetyl-6-*O*-benzyl-α-D-glucopyranosyl)-(1→4)-2,3,6-tri-*O*-acetyl-β-D-glucopyranoside (8):** To a solution of compound **2** (1.2 g, 1.60 mmol) and compound **7** (1.6 g, 1.89 mmol) in anhydrous CH_2_Cl_2_ (8 mL) MS 4Å (2.0 g) was added and the reaction mixture was cooled to −10 °C. To the cooled reaction mixture NIS (500.0 mg, 2.22 mmol) and HClO_4_–SiO_2_ (20.0 mg) were added and it was allowed to stir for 1 h at same temperature. The reaction mixture was filtered through a bed of Celite^®^ and washed with CH_2_Cl_2_ (100 mL). The organic layer was successively washed with 5% aq. Na_2_S_2_O_3_, NaHCO_3_ solution and water, dried (Na_2_SO_4_) and concentrated under reduced pressure. The crude product was purified over silica gel using hexane–EtOAc (2:1) as eluant to give pure compound **8** (2.0 g, 82%). White solid; mp 66–68 °C; [α]_D_^25^ +18.2 (*c* 1.2, CHCl_3_); IR (KBr): 3428, 3032, 2932, 2108, 1753, 1508, 1455, 1368, 1230, 1044, 740, 698 cm^−1^; ^1^H NMR (600 MHz, CDCl_3_) δ 7.35–7.05 (m, 30H, Ar-H), 6.91 (d, *J* = 9.0 Hz, 2H, Ar-H), 6.80 (*J* = 9.0 Hz, 2H, Ar-H), 5.34 (d, *J* = 4.5 Hz, 1H, H-1_B_), 5.29 (t, *J* = 10.0 Hz, 1H, H-3_A_), 5.27 (t, *J* = 9.0 Hz, 1H, H-3_B_), 5.04 (dd, *J* = 8.0 Hz each, 1H, H-2_A_), 4.97 (d, *J* = 3.5 Hz, 1H, H-1_D_), 4.95 (d, *J* = 8.0 Hz, 1H, H-1_A_), 4.89 (d, *J* = 2.0 Hz, 1H, H-1_C_), 4.85–4.80 (m, 2H, PhC*H*_2_), 4.78–4.75 (m, 3H, H-2_B_, PhC*H*_2_), 4.65 (d, *J* = 12.0 Hz, 1H, PhC*H*_2_), 4.60 (d, *J* = 12.0 Hz, 1H, PhC*H*_2_), 4.58–4.44 (m, 5H, PhC*H*_2_), 4.41 (dd, *J* = 12.5, 2.5 Hz, 1H, H-6_aA_), 4.35 (d, *J* = 12.0 Hz, 1H, PhC*H*_2_), 4.21 (dd, *J* = 12.5, 2.5 Hz, 1H, H-6_bA_), 4.05–4.01 (2 t, *J* = 9.5 Hz each, 2H, H-3_D_, H-4_C_), 3.98–3.92 (m, 2H, H-3c, H-5c), 3.90 (t, *J* = 10.0 Hz, 1H, H-4_D_), 3.81 (t, *J* = 8.5 Hz, 1H, H-4_A_), 3.77 (s, 3H, OC*H*_3_), 3.76–3.74 (m, 1H, H-4_B_), 3.72–3.71 (m, 1H, H-5_B_), 3.69–3.65 (m, 2H, H-5_A_, H-6_aB_), 3.63–3.61 (m, 1H, H-6_aD_), 3.58–3.55 (m, 3H, H-2_C_, H-5_D_, H-6_bB_), 3.53–3.51 (m, 1H, H-6_bD_), 3.43 (dd, *J* = 10.0, 3.5 Hz, 1H, H-2_D_), 2.04, 2.02, 2.01, 1.99, 1.95 (5 s, 15H, 5 COC*H*_3_), 1.25 (d, *J* = 6.2 Hz, 3H, CC*H*_3_); ^13^C NMR (150 MHz, CDCl_3_) δ 170.7, 170.2 (2C), 169.8, 169.7 (5 *C*OCH_3_), 155.7–114.5 (Ar-C), 99.7 (C-1_A_), 98.8 (C-1_C_), 96.0 (C-1_B_), 93.9 (C-1_D_), 80.2 (C-3_D_), 79.4 (C-4_D_), 78.2 (C-5_A_), 77.2 (C-5_B_), 75.5 (Ph*C*H_2_), 75.4 (C-3_A_), 75.3 (Ph*C*H_2_), 74.8 (Ph*C*H_2_), 74.3 (C-2_B_), 74.2 (C-4_C_), 73.7 (Ph*C*H_2_), 73.3 (Ph*C*H_2_), 72.6 (2C, C-5_D_, Ph*C*H_2_), 72.2 (C-4_A_), 72.1 (C-2_A_), 71.3 (C-4_B_), 70.7 (2C, C-3_C_, C-5_C_), 70.4 (C-3_B_), 69.0 (C-2_C_), 67.9 (C-6_B_), 67.8 (C-6_D_), 63.3 (C-2_D_), 62.6 (C-6_A_), 55.6 (O*C*H_3_), 21.0, 20.9, 20.7, 20.6 (2C) (5 CO*C*H_3_), 17.9 (C*C*H_3_); ESIMS *m*/*z*: 1554.6 [M + Na]^+^; anal. calcd for C_83_H_93_N_3_O_25_ (1531.61): C, 65.04; H, 6.12; found: C, 64.82; H, 6.36.

**4-Methoxyphenyl (2-acetamido-2-deoxy-α-D-glucopyranosyl)-(1→3)-(α-L-rhamnopyranosyl)-(1→4)-(α-D-glucopyranosyl)-(1→4)-sodium β-D-glucopyranosid uronate (1):** A solution of compound **8** (1.3 g, 0.85 mmol) in 0.1 M CH_3_ONa in CH_3_OH (25 mL) was allowed to stir at rt for 3 h and neutralized with Dowex 50W X8 (H^+^) resin. The reaction mixture was filtered and concentrated under reduced pressure. To a solution of the crude product in CH_2_Cl_2_ (25 mL) and H_2_O (4 mL) were sequentially added aq. NaBr (2 mL, 1 M), aq. TBAB (2.5 mL, 1 M), TEMPO (100.0 mg, 0.64 mmol), satd. NaHCO_3_ solution (10 mL) and 4% aq. NaOCl (15 mL) were added and the reaction mixture was allowed to stir at 0–5 °C for 2 h. The reaction mixture was neutralized with 1 N aq. HCl. To the reaction mixture *tert*-butanol (25 mL), 2-methylbut-2-ene (20 mL, 2 M solution in THF), aq. NaClO_2_ (1.5 g in 5 mL) and aq. NaH_2_PO_4_ (1.5 g in 5 mL) were added and the reaction mixture was stirred at room temperature for 3 h. The reaction mixture was diluted with satd. aq. NaH_2_PO_4_ and extracted with CH_2_Cl_2_ (150 mL). The organic layer was washed with water, dried (Na_2_SO_4_) and concentrated to dryness. The crude product was passed through a short pad of silica gel using EtOAc–toluene (2:1) as eluant. To a solution of the oxidized product (800.0 mg) in CH_3_OH–EtOAc (20 mL, 10:1 *v*/*v*) 20% Pd(OH)_2_/C (150.0 mg) was added and the reaction mixture was allowed to stir at room temperature under a positive pressure of hydrogen for 10 h. The reaction mixture was filtered through a bed of Celite^®^ and evaporated to dryness. To a solution of the crude product in CH_3_OH (10 mL) acetic anhydride (2 mL) was added and the solution was kept at rt for 30 min. The solvents were removed under reduced pressure and the product was passed through a Sephadex^®^ LH-20 column using CH_3_OH–H_2_O (60 mL, 4:1 v/v) as eluant to give pure compound **1** (450.0 mg, 64%). White powder; [α]_D_^25^ +14 (*c* 1.0, CH_3_OH); IR (KBr): 3428, 2937, 1621, 1366, 1152, 1087, 669 cm^−1^; ^1^H NMR (600 MHz, CD_3_OD) δ 7.04 (d, *J* = 9.0 Hz, 2H, Ar-H), 6.84 (d, *J* = 9.0 Hz, 2H, Ar-H), 5.20 (d, *J* = 3.6 Hz, 1H, H-1_B_), 4.93 (br s, 1H, H-1_D_), 4.89 (br s, 1H, H-1_C_), 4.82 (d, *J* = 7.8 Hz, 1H, H-1_A_), 4.03–3.98 (m, 2H, H-2_A_, H-5_C_), 3.97–3.95 (m, 2H, H-2_C_, H-2_D_), 3.88–3.86 (m, 1H, H-6_aD_), 3.83–3.75 (m, 5H, H-3_A_, H-3_B_, H-5_D_, H-6_aB_, H-6_bD_), 3.74 (s, 3H, OC*H*_3_), 3.73–3.71 (m, 1H, H-4_A_), 3.70–3.67 (m, 2H, H-2_B_, H-6_bB_), 3.64 (t, *J* = 9.6 Hz, 1H, H-3_D_), 3.56–3.50 (m, 4H, H-4_B_, H-4_C_, H-5_A_, H-5_B_), 3.49–3.44 (m, 2H, H-3_C_, H-4_D_), 2.02 (s, 3H, COC*H*_3_), 1.30 (d, *J* = 6.1Hz, 3H, CC*H*_3_); ^13^C NMR (150 MHz, CDCl_3_) δ 175.0 (*C*OONa), 172.6 (NH*C*OCH_3_), 156.7–115.5 (Ar-C), 103.3 (C-1_A_), 102.8 (C-1_B_), 102.4 (C-1_C_), 96.7 (C-1_D_), 81.2 (C-3_D_), 79.5 (C-4_B_), 78.1 (C-3_A_), 77.7 (C-4_A_), 76.6 (C-5_A_), 74.6 (C-3_C_), 74.2 (C-4_C_), 73.8 (C-2_B_), 73.6 (C-2_A_), 73.0 (C-5_D_), 72.0 (C-5_B_), 71.9 (C-4_D_), 70.8 (C-5_C_), 69.3 (C-2_C_), 62.1 (C-6_B_), 62.0 (C-6_D_), 56.2 (O*C*H_3_), 55.3 (C-2_D_), 23.1 (NHCO*C*H_3_), 18.1 (C*C*H_3_); ESIMS *m*/*z*: 834.2 [M + H]^+^; anal. calcd for C_33_H_48_NNaO_22_ (833.26): C, 47.54; H, 5.80; found: C, 47.72; H, 6.07.

## Supporting Information

File 1 1D and 2D NMR spectra of compounds **2**, **6**, **7**, **8** and **1**.
