# Supplementary material for: Convergent synthesis of the tetrasaccharide repeating unit of the O-antigen of Shigella boydii type 9
Source: Beilstein J Org Chem. 2011 Aug 29;7:1182–8. doi: 10.3762/bjoc.7.137 (PMC3182426; doi:10.3762/bjoc.7.137)
Supplement: File 1 — 1D and 2D NMR spectra of compounds 2, 6, 7, 8 and 1. [file Beilstein_J_Org_Chem-07-1182-s001.pdf]

Supporting Information

for

Convergent synthesis of the tetrasaccharide repeating unit of the *O*-antigen of *Shigella boydii* type 9

Abhishek Santra and Anup Kumar Misra*

Address: Bose Institute, Division of Molecular Medicine, P-1/12, C.I.T. Scheme VII-M, Kolkata-700054, India; Fax: 91-33-2355 3886

Email: Anup Kumar Misra* - akmisra69@gmail.com

* Corresponding author

1D and 2D NMR spectra of compounds 2, 6, 7, 8 and 1

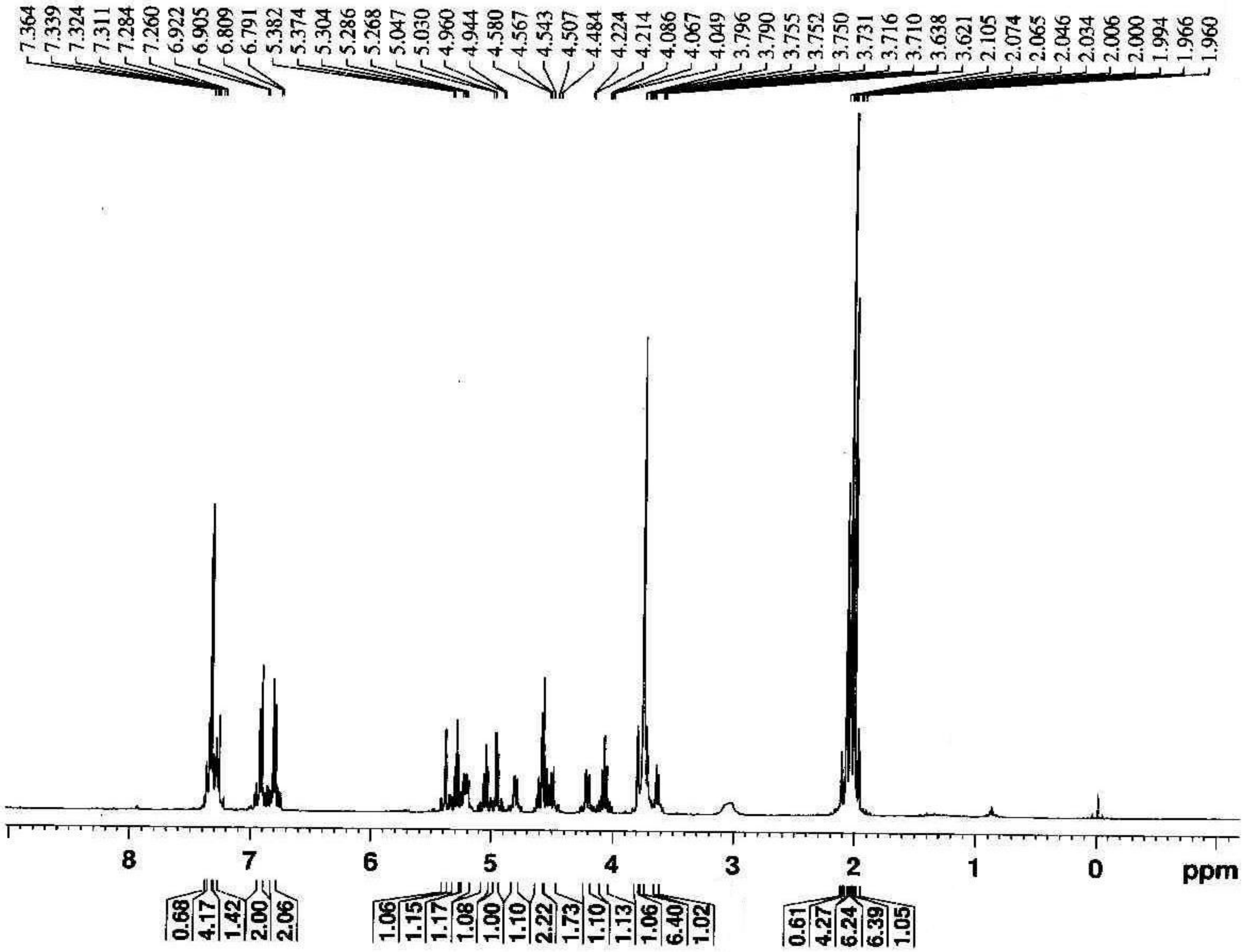

^{1}H NMR spectrum of 4-methoxyphenyl (2,3-di-*O*-acetyl-6-*O*-benzyl-α-D-glucopyranosyl)-(1→4)-2,3,6-tri-*O*-acetyl-β-D-glucopyranoside (**2**).

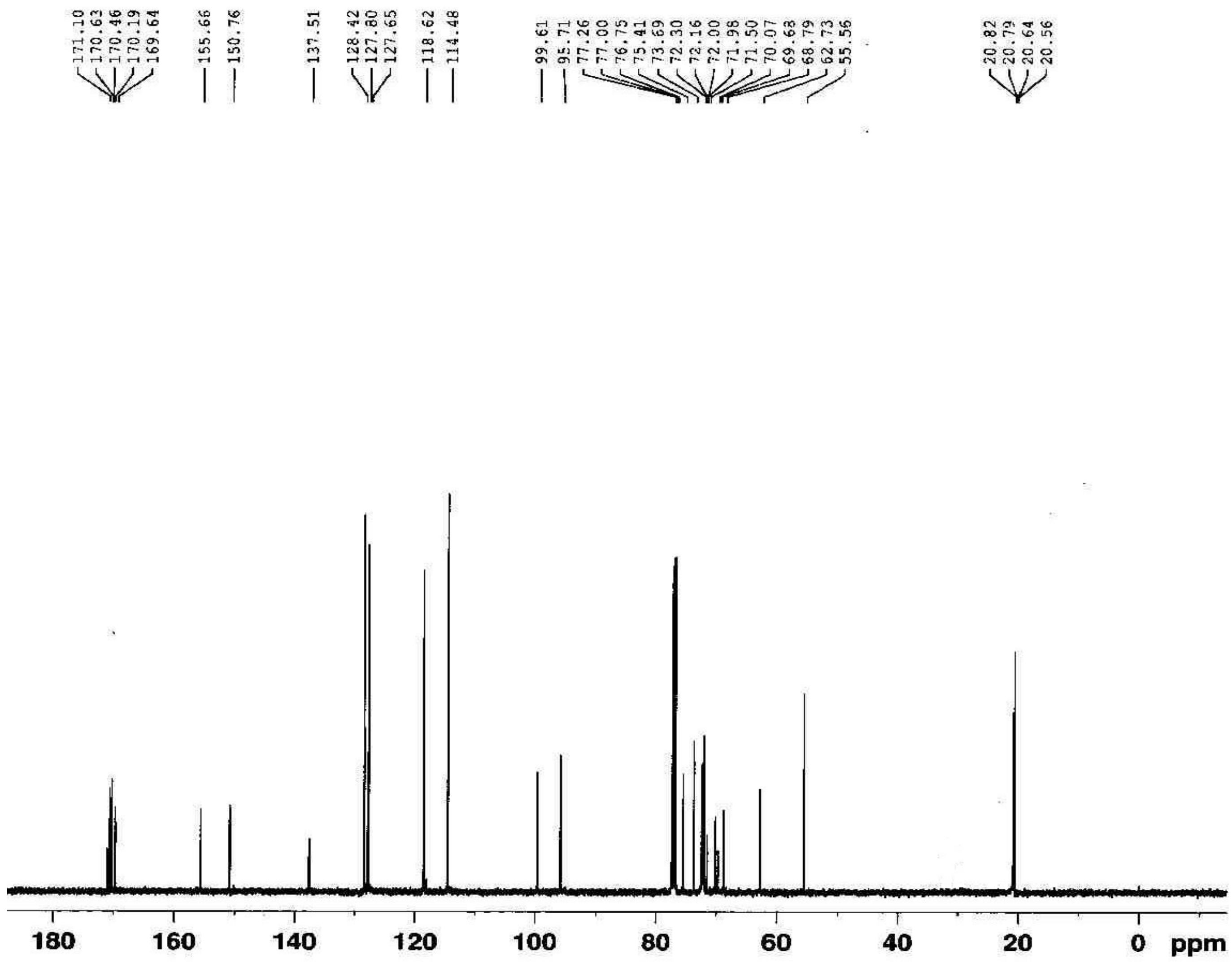

^{13}C NMR spectrum of 4-methoxyphenyl (2,3-di-*O*-acetyl-6-*O*-benzyl-α-D-glucopyranosyl)-(1→4)-2,3,6-tri-*O*-acetyl-β-D-glucopyranoside (**2**).

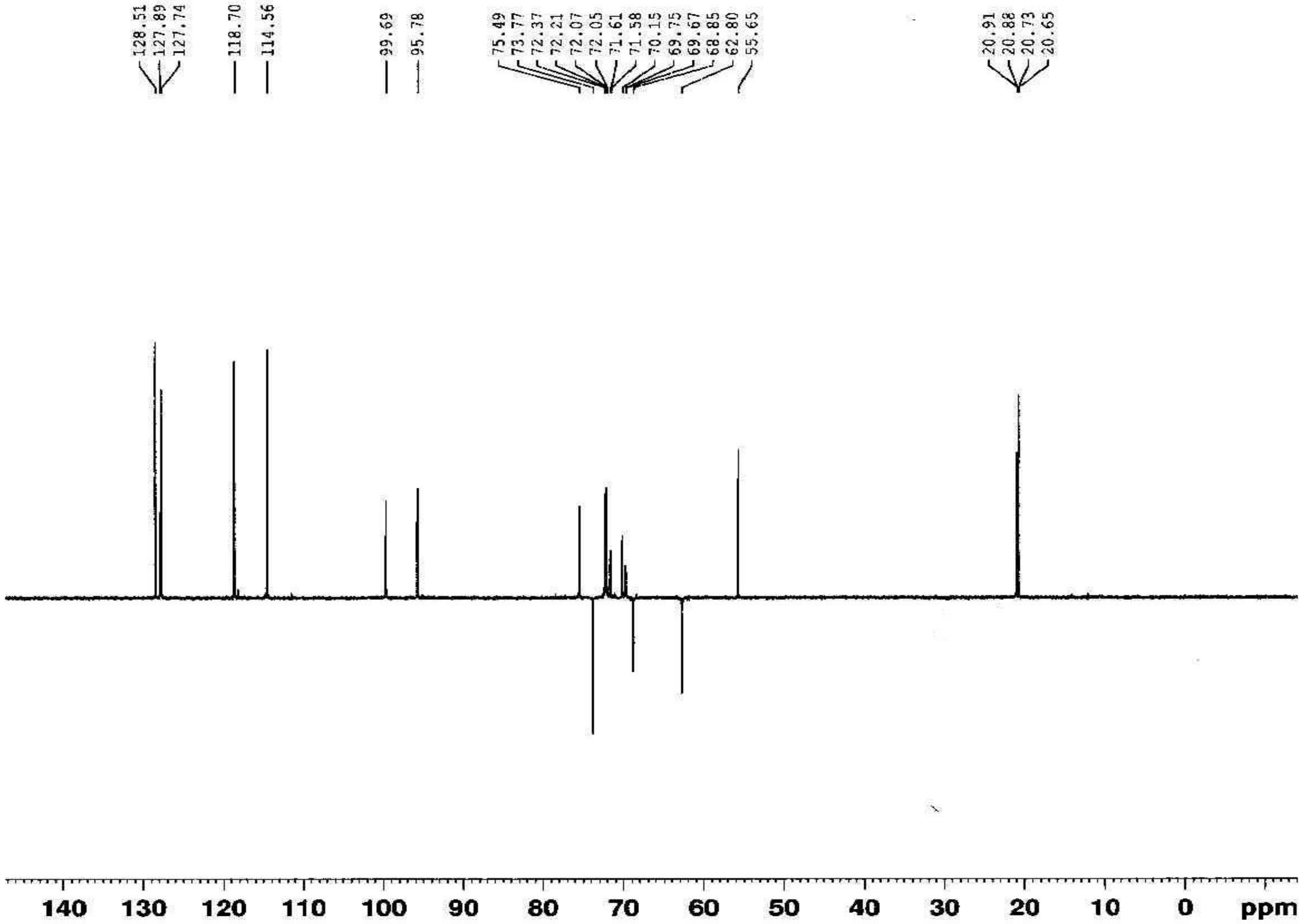

^{13}C DEPT 135 NMR spectrum of 4-methoxyphenyl (2,3-di-*O*-acetyl-6-*O*-benzyl-α-D-glucopyranosyl)-(1→4)-2,3,6-tri-*O*-acetyl-β-D-glucopyranoside (**2**).

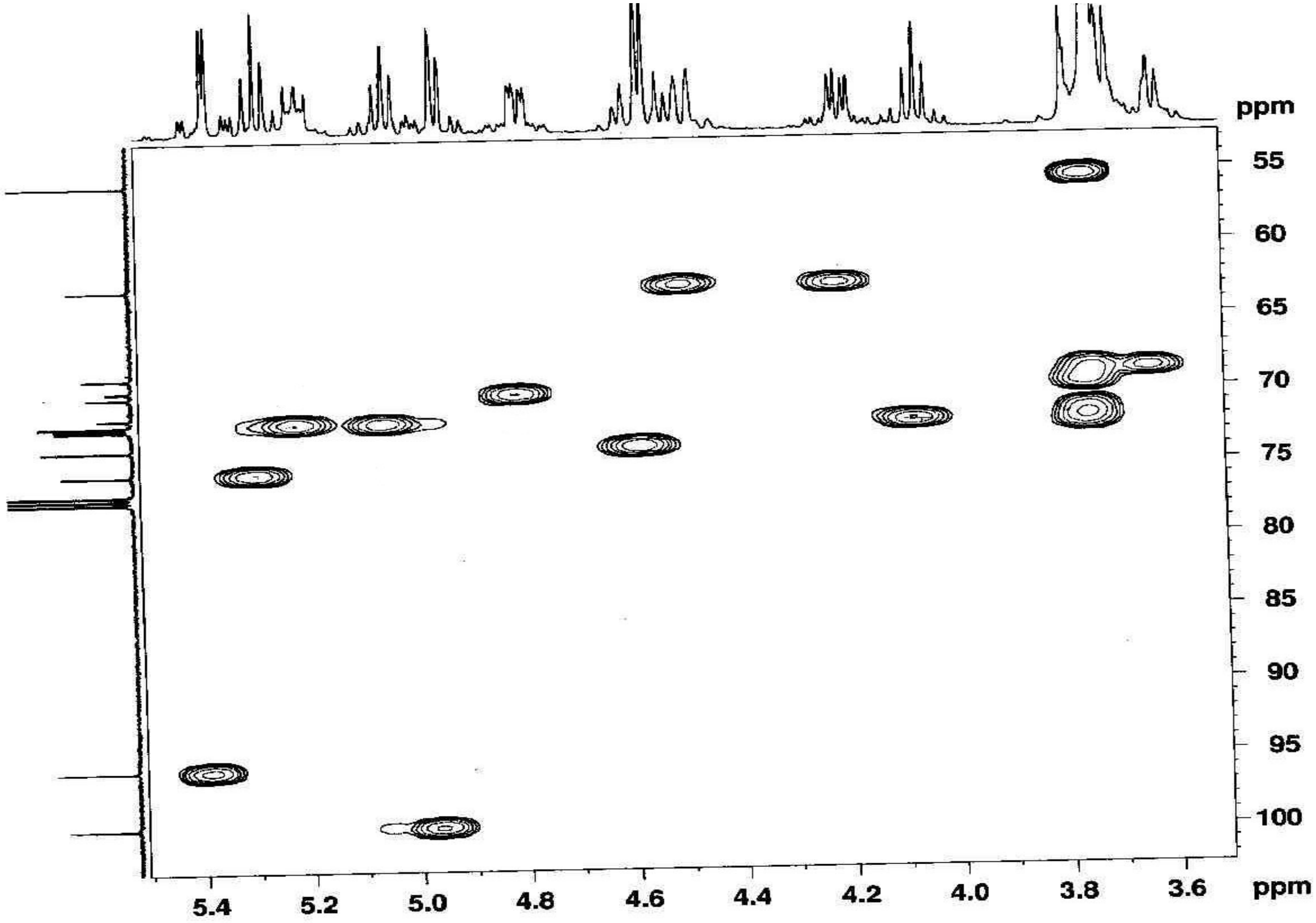

2D HMQC NMR spectrum of 4-methoxyphenyl (2,3-di-*O*-acetyl-6-*O*-benzyl-α-D-glucopyranosyl)-(1→4)-2,3,6-tri-*O*-acetyl-β-D-glucopyranoside (**2**) (expanded region).

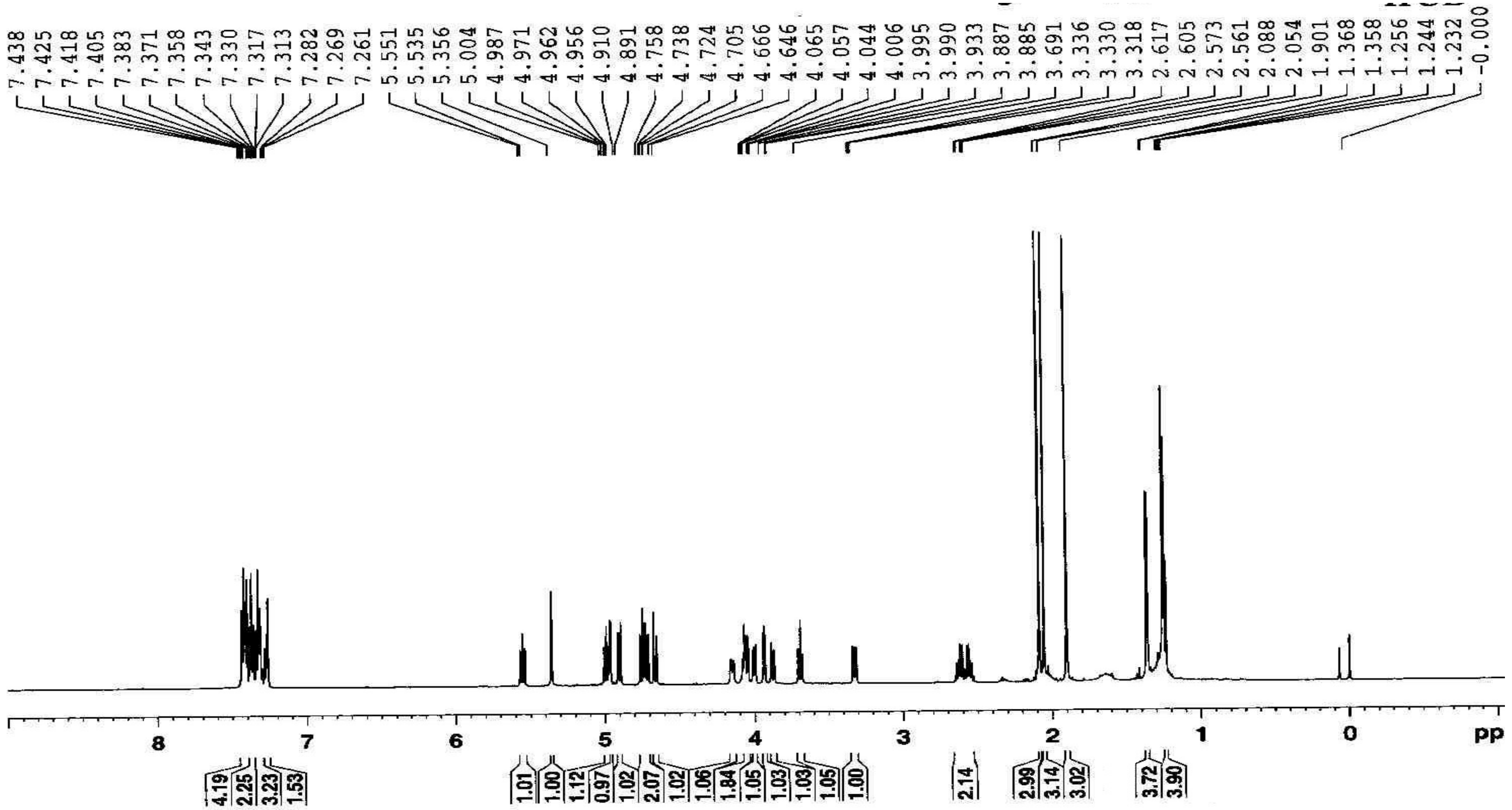

^{1}H NMR spectrum of ethyl (3,4,6-tri-*O*-acetyl-2-azido-2-deoxy-α-D-glucopyranosyl)-(1→3)-2,4-di-*O*-benzyl-1-thio-α-L-rhamnopyranoside (**6**).

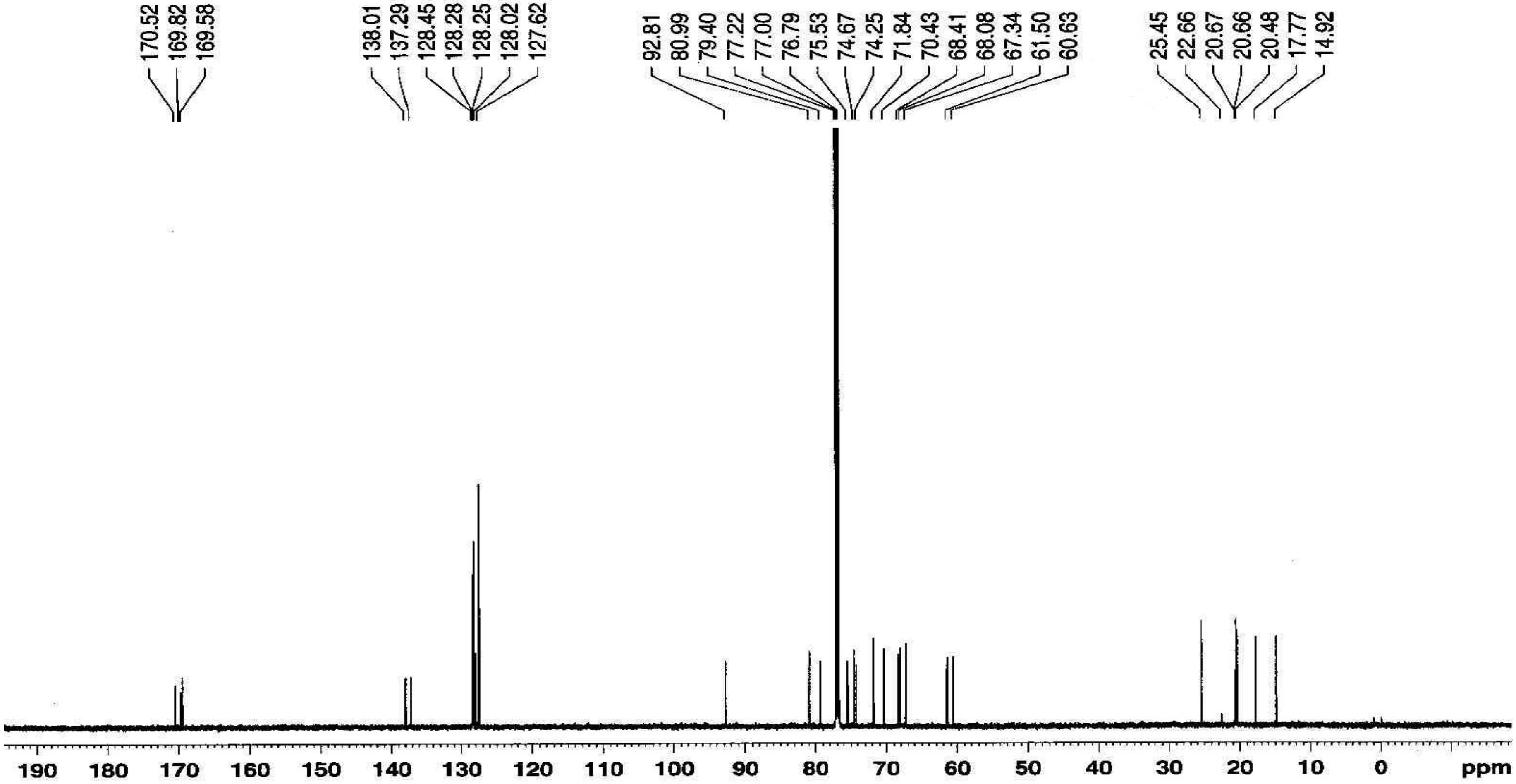

^{13}C NMR spectrum of ethyl (3,4,6-tri-*O*-acetyl-2-azido-2-deoxy-α-D-glucopyranosyl)-(1→3)-2,4-di-*O*-benzyl-1-thio-α-L-rhamnopyranoside (**6**).

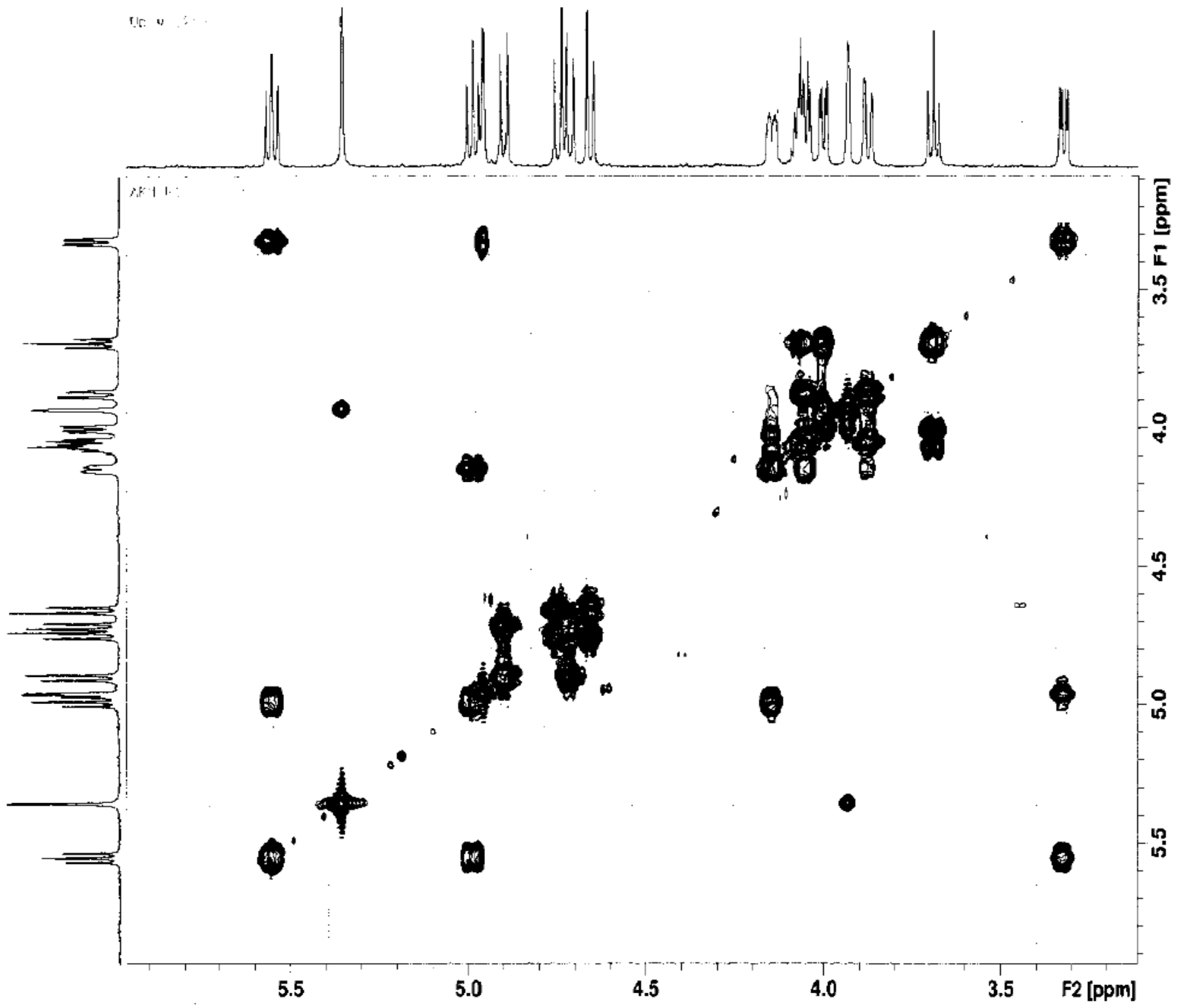

2D COSY NMR spectrum of ethyl (3,4,6-tri-*O*-acetyl-2-azido-2-deoxy-α-D-glucopyranosyl)-(1→3)-2,4-di-*O*-benzyl-1-thio-α-L-rhamnopyranoside (**6**) (expanded region).

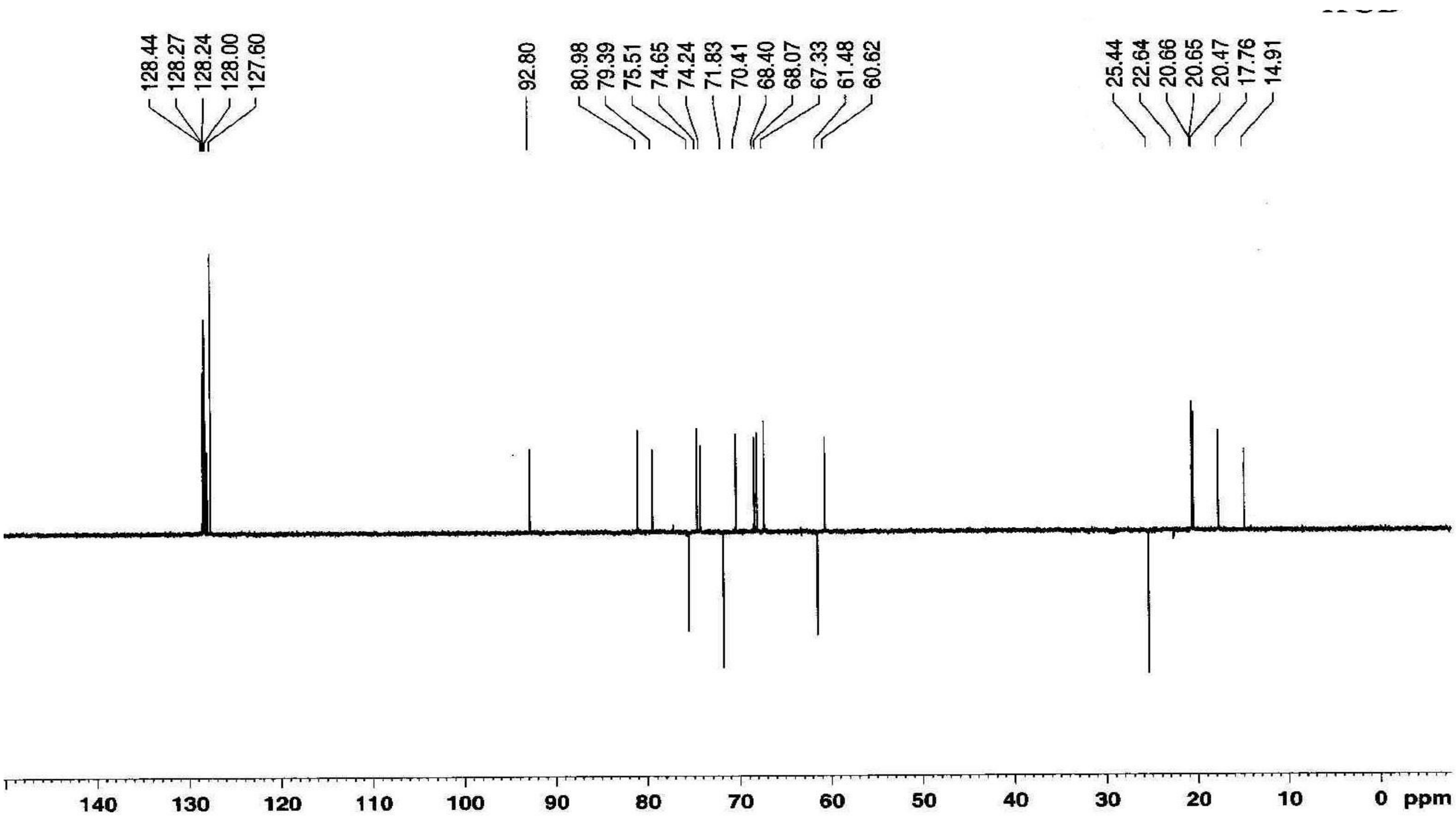

^{13}C DEPT 135 NMR spectrum of ethyl (3,4,6-tri-*O*-acetyl-2-azido-2-deoxy-α-D-glucopyranosyl)-(1→3)-2,4-di-*O*-benzyl-1-thio-α-L-rhamnopyranoside (**6**).

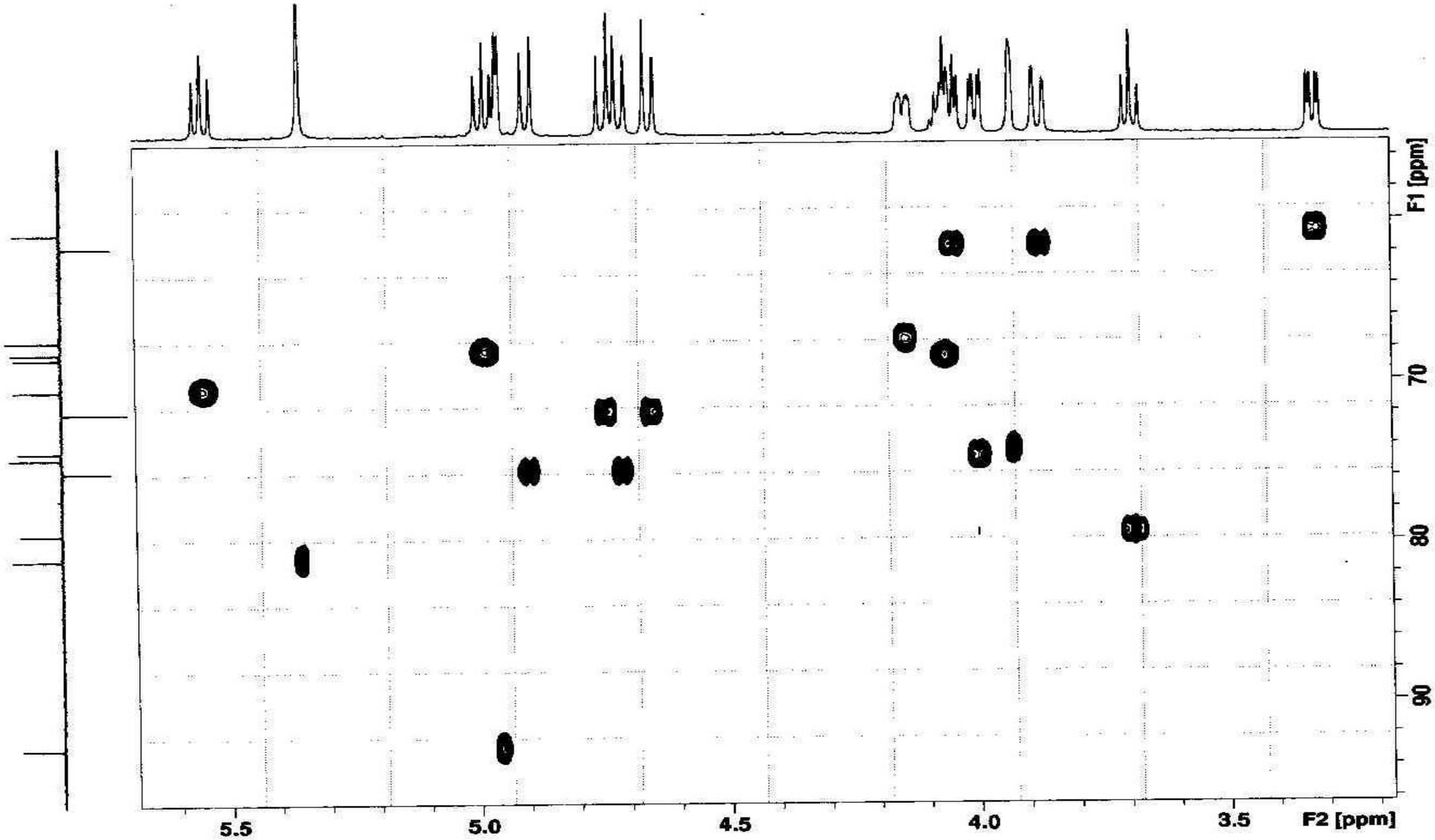

2D HMQC NMR spectrum of ethyl (3,4,6-tri-*O*-acetyl-2-azido-2-deoxy-α-D-glucopyranosyl)-(1→3)-2,4-di-*O*-benzyl-1-thio-α-L-rhamnopyranoside (**6**) (expanded region).

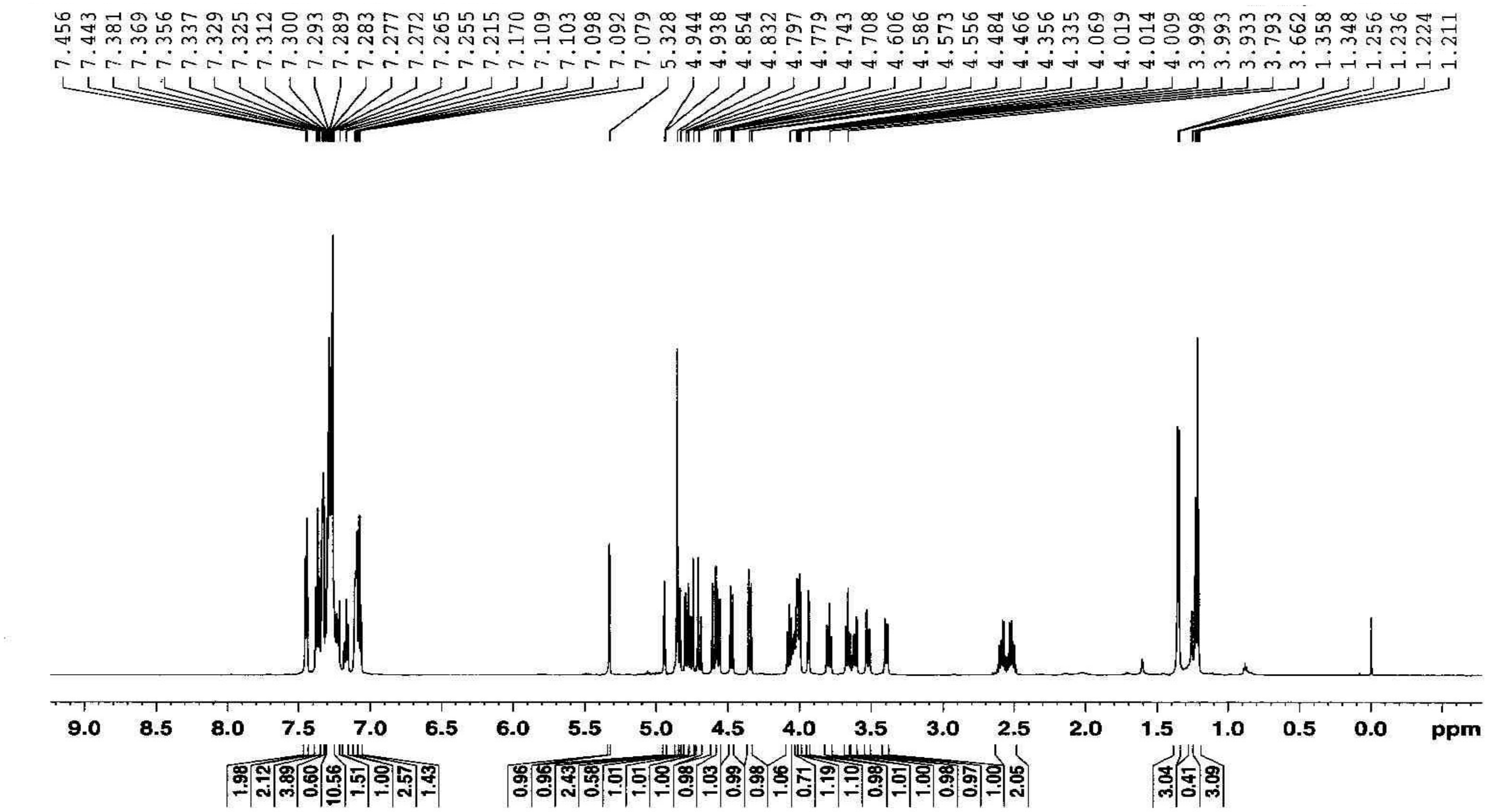

^{1}H NMR spectrum of ethyl (2-azido-3,4,6-tri-*O*-benzyl-2-deoxy-α-D-glucopyranosyl)-(1→3)-2,4-di-*O*-benzyl-1-thio-α-L-rhamnopyranoside (**7**).

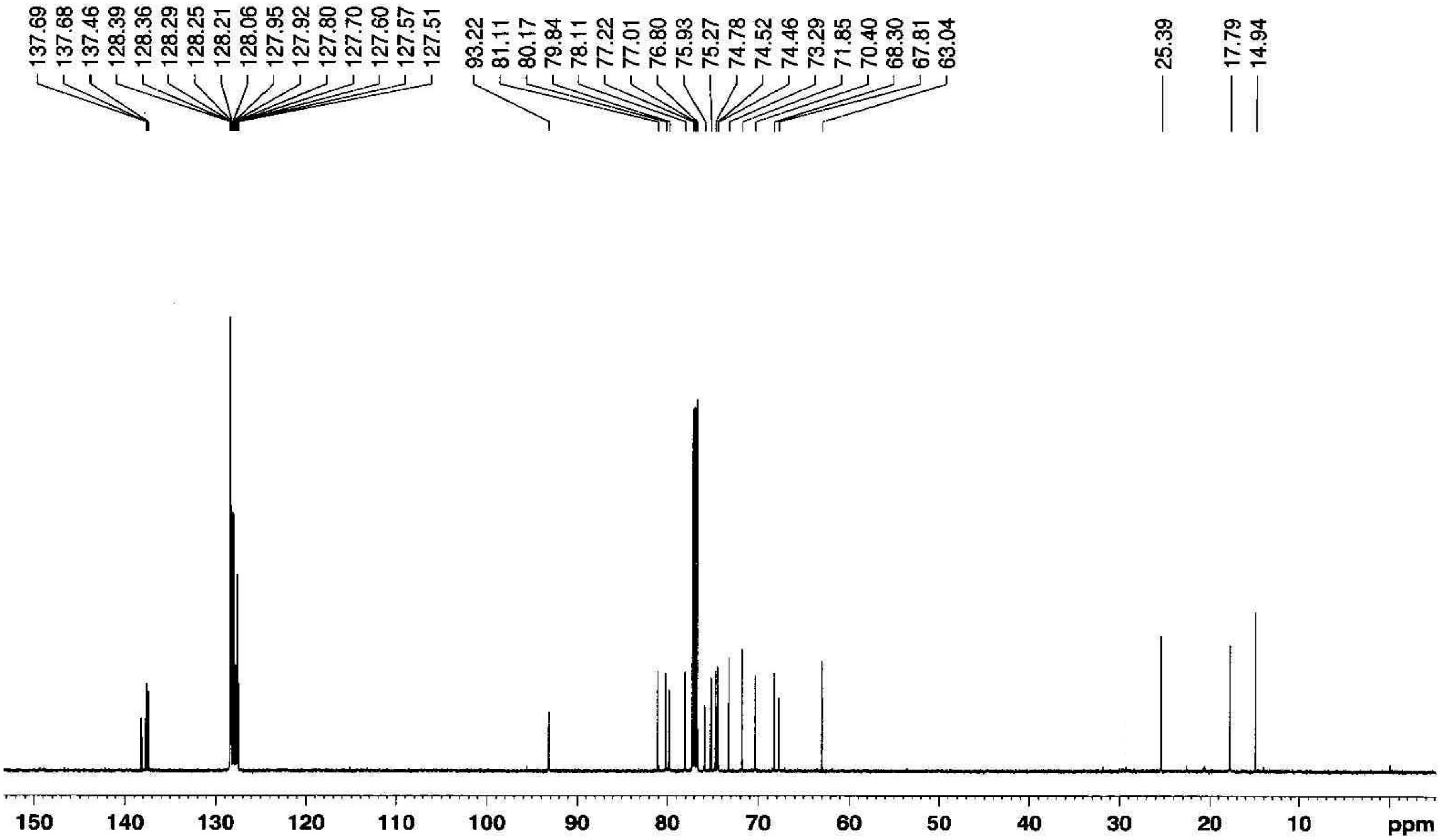

^{13}C NMR spectrum of ethyl (2-azido-3,4,6-tri-*O*-benzyl-2-deoxy-α-D-glucopyranosyl)-(1→3)-2,4-di-*O*-benzyl-1-thio-α-L-rhamnopyranoside (**7**).

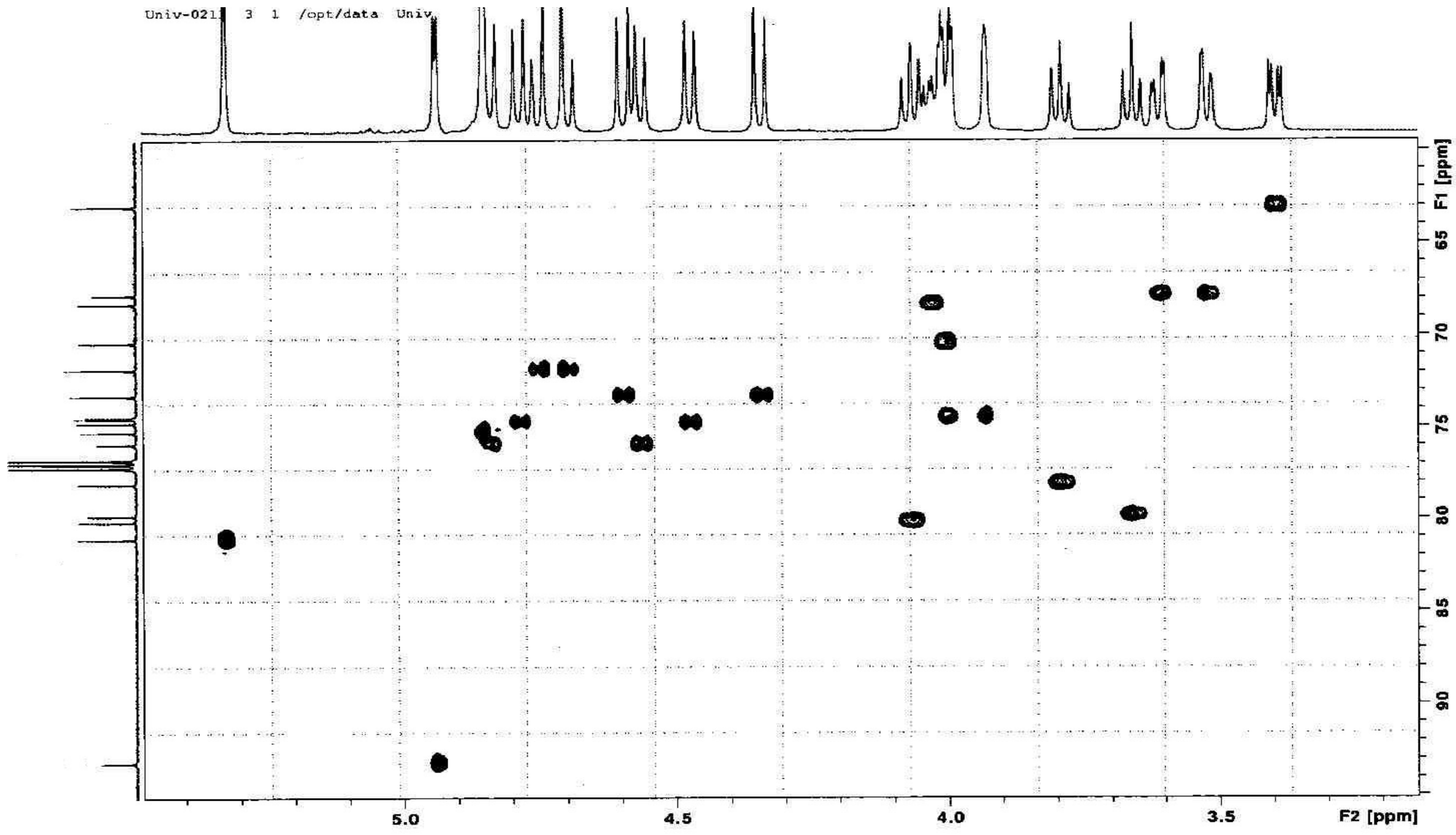

2D HMQC NMR spectrum of ethyl (2-azido-3,4,6-tri-*O*-benzyl-2-deoxy-α-D-glucopyranosyl)-(1→3)-2,4-di-*O*-benzyl-1-thio-α-L-rhamnopyranoside (**7**) (expanded region).

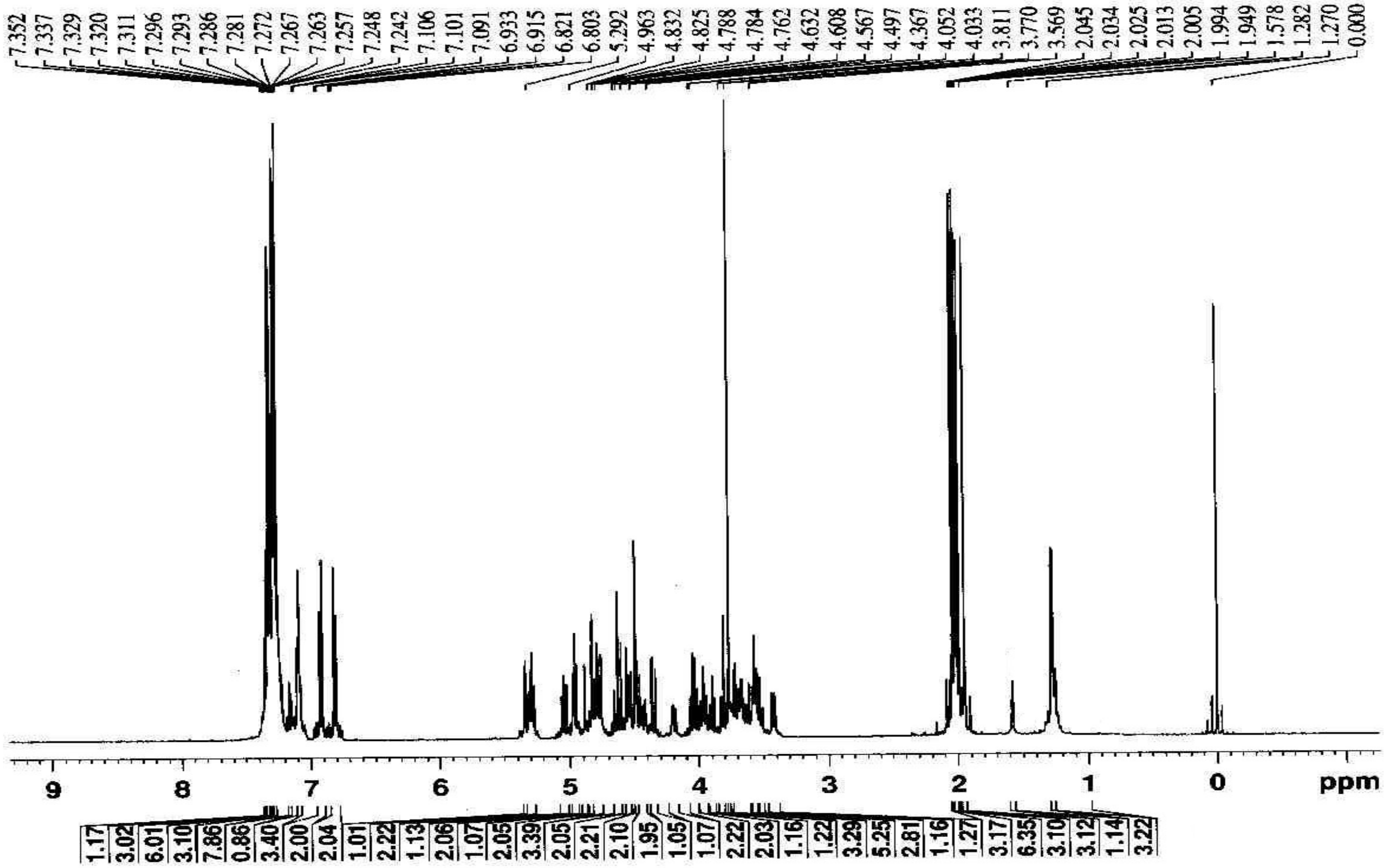

^{1}H NMR spectrum of 4-methoxyphenyl (2-azido-3,4,6-tri-*O*-benzyl-2-deoxy-α-D-glucopyranosyl)-(1→3)-(2,4-di-*O*-benzyl-α-L-rhamnopyranosyl)-(1→4)-(2,3-di-*O*-acetyl-6-*O*-benzyl-α-D-glucopyranosyl)-(1→4)-2,3,6-tri-*O*-acetyl-β-D-glucopyranoside (**8**).

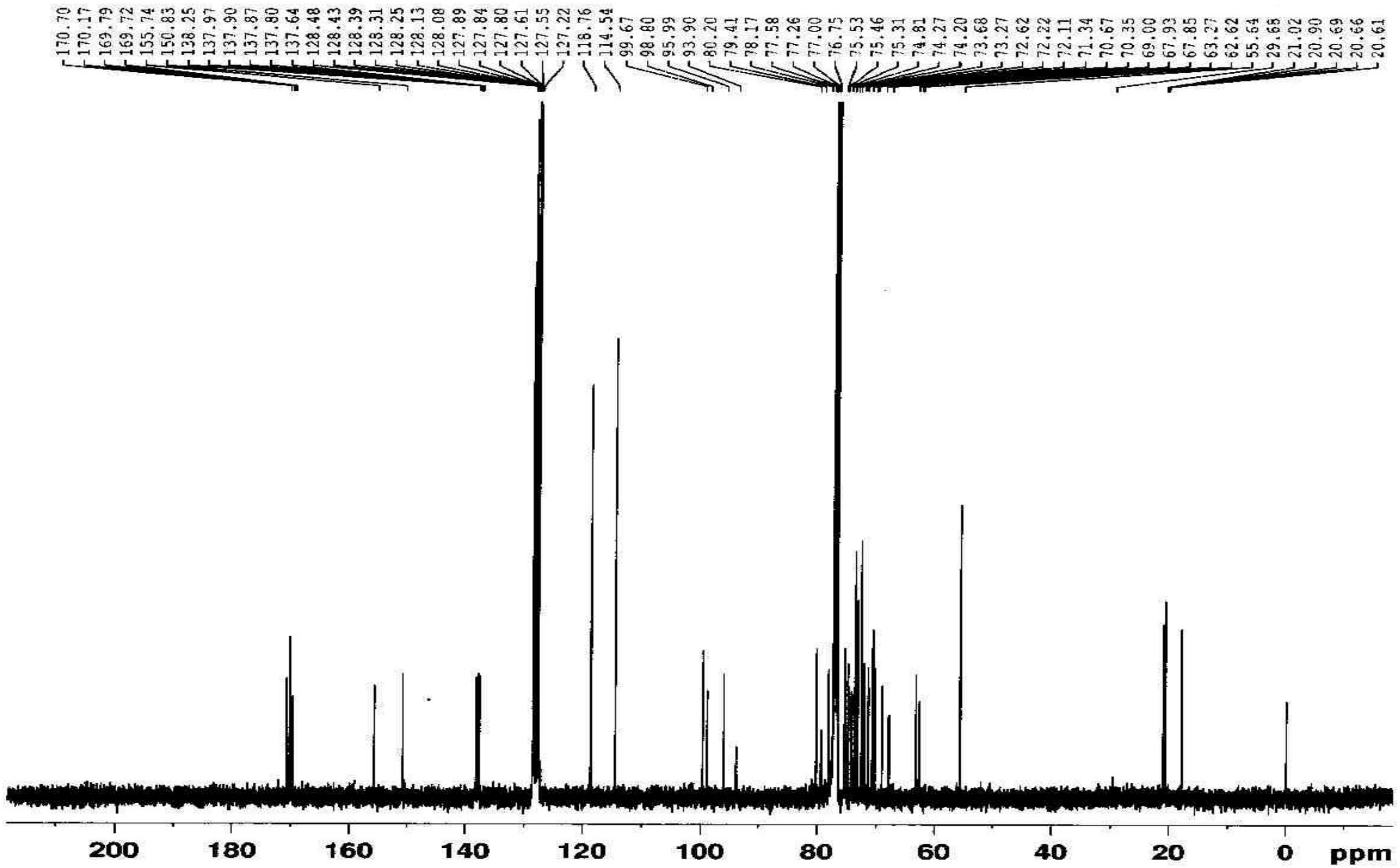

^{13}C NMR spectrum of 4-methoxyphenyl (2-azido-3,4,6-tri-*O*-benzyl-2-deoxy-α-D-glucopyranosyl)-(1→3)-(2,4-di-*O*-benzyl-α-L-rhamnopyranosyl)-(1→4)-(2,3-di-*O*-acetyl-6-*O*-benzyl-α-D-glucopyranosyl)-(1→4)-2,3,6-tri-*O*-acetyl-β-D-glucopyranoside (**8**).

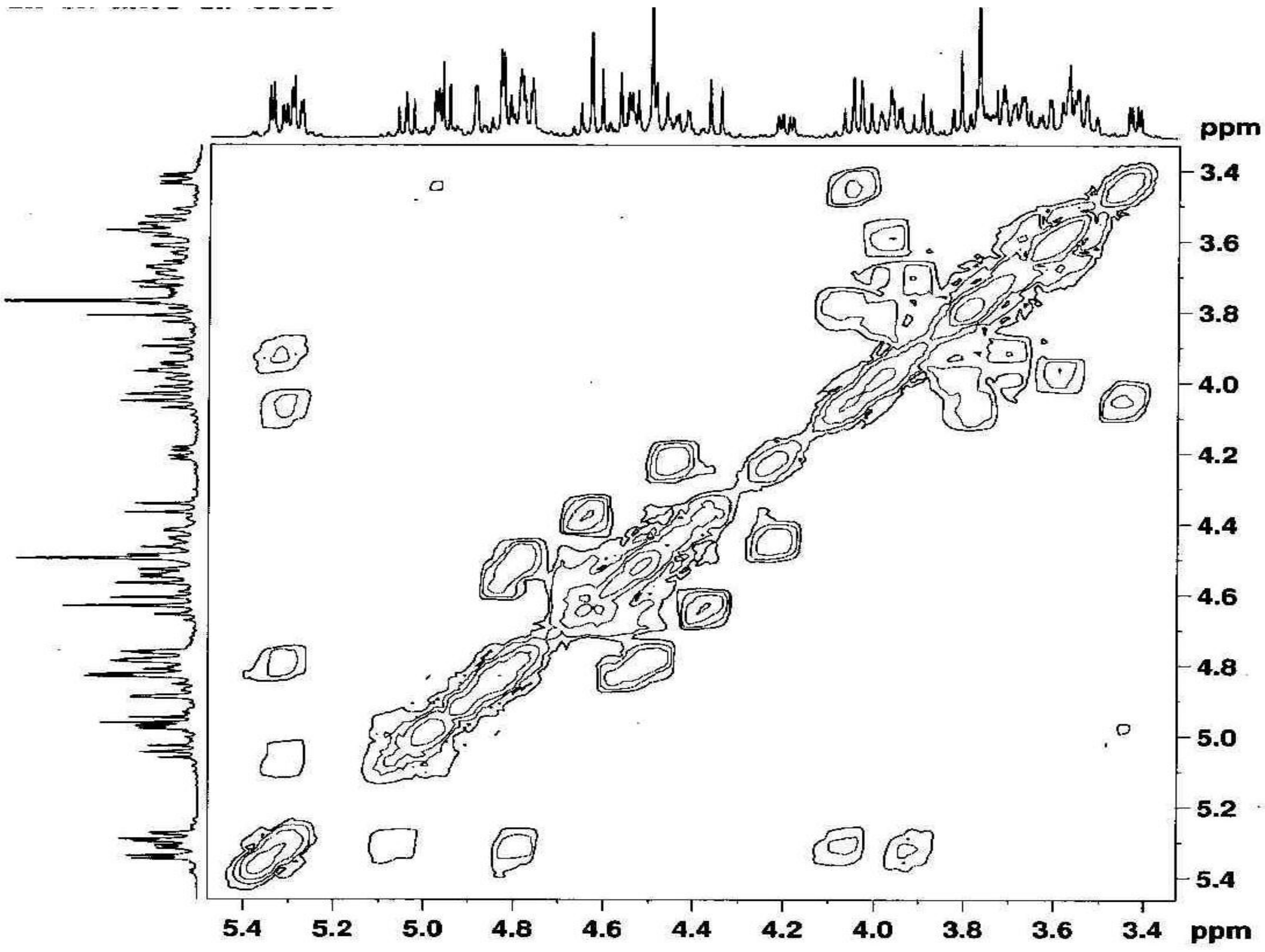

2D COSY NMR spectrum of 4-methoxyphenyl (2-azido-3,4,6-tri-*O*-benzyl-2-deoxy-α-D-glucopyranosyl)-(1→3)-(2,4-di-*O*-benzyl-α-L-rhamnopyranosyl)-(1→4)-(2,3-di-*O*-acetyl-6-*O*-benzyl-α-D-glucopyranosyl)-(1→4)-2,3,6-tri-*O*-acetyl-β-D-glucopyranoside (**8**) (expanded region).

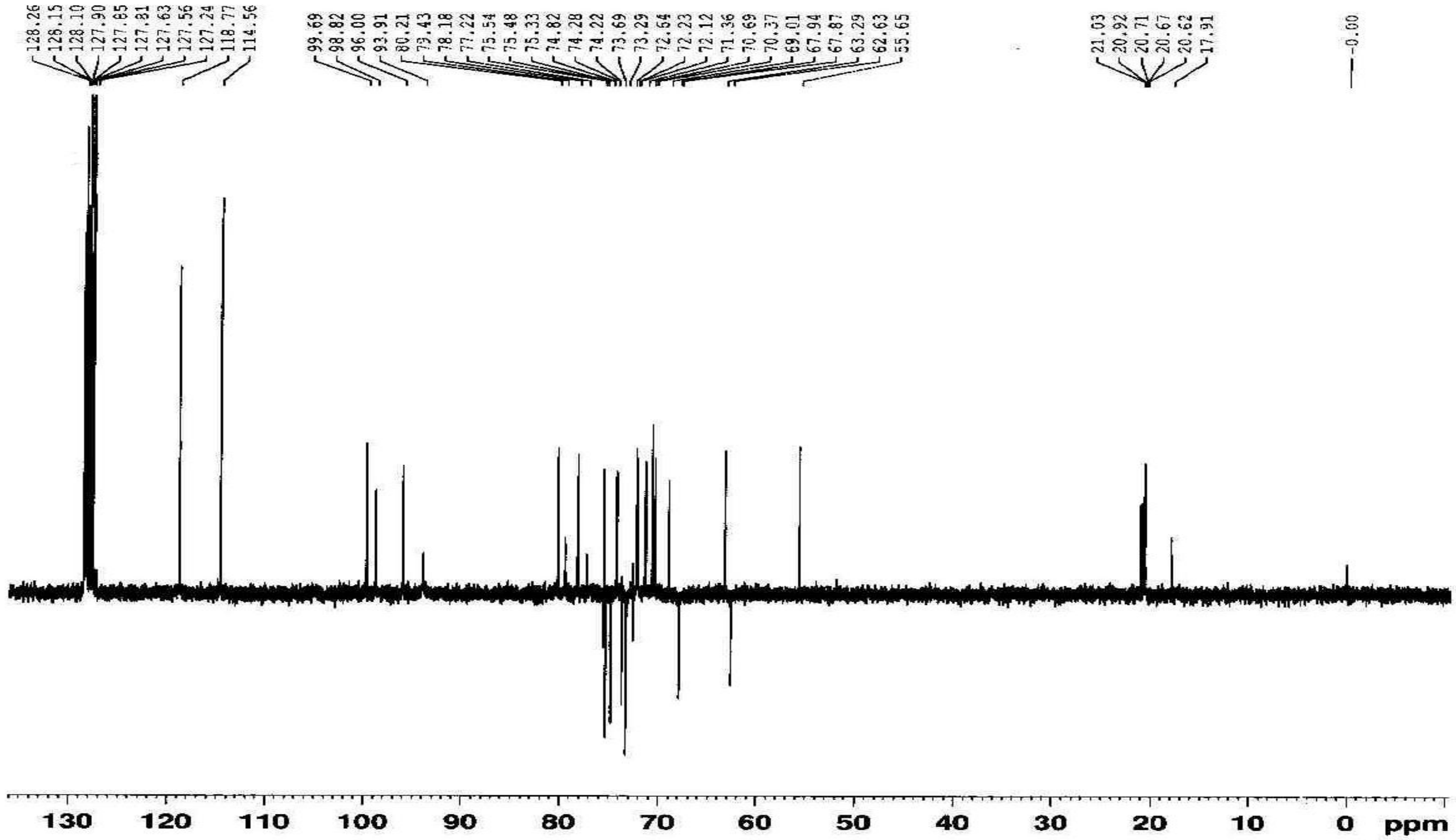

^{13}C DEPT 135 NMR spectrum of 4-methoxyphenyl (2-azido-3,4,6-tri-*O*-benzyl-2-deoxy-α-D-glucopyranosyl)-(1→3)-(2,4-di-*O*-benzyl-α-L-rhamnopyranosyl)-(1→4)-(2,3-di-*O*-acetyl-6-*O*-benzyl-α-D-glucopyranosyl)-(1→4)-2,3,6-tri-*O*-acetyl-β-D-glucopyranoside (**8**).

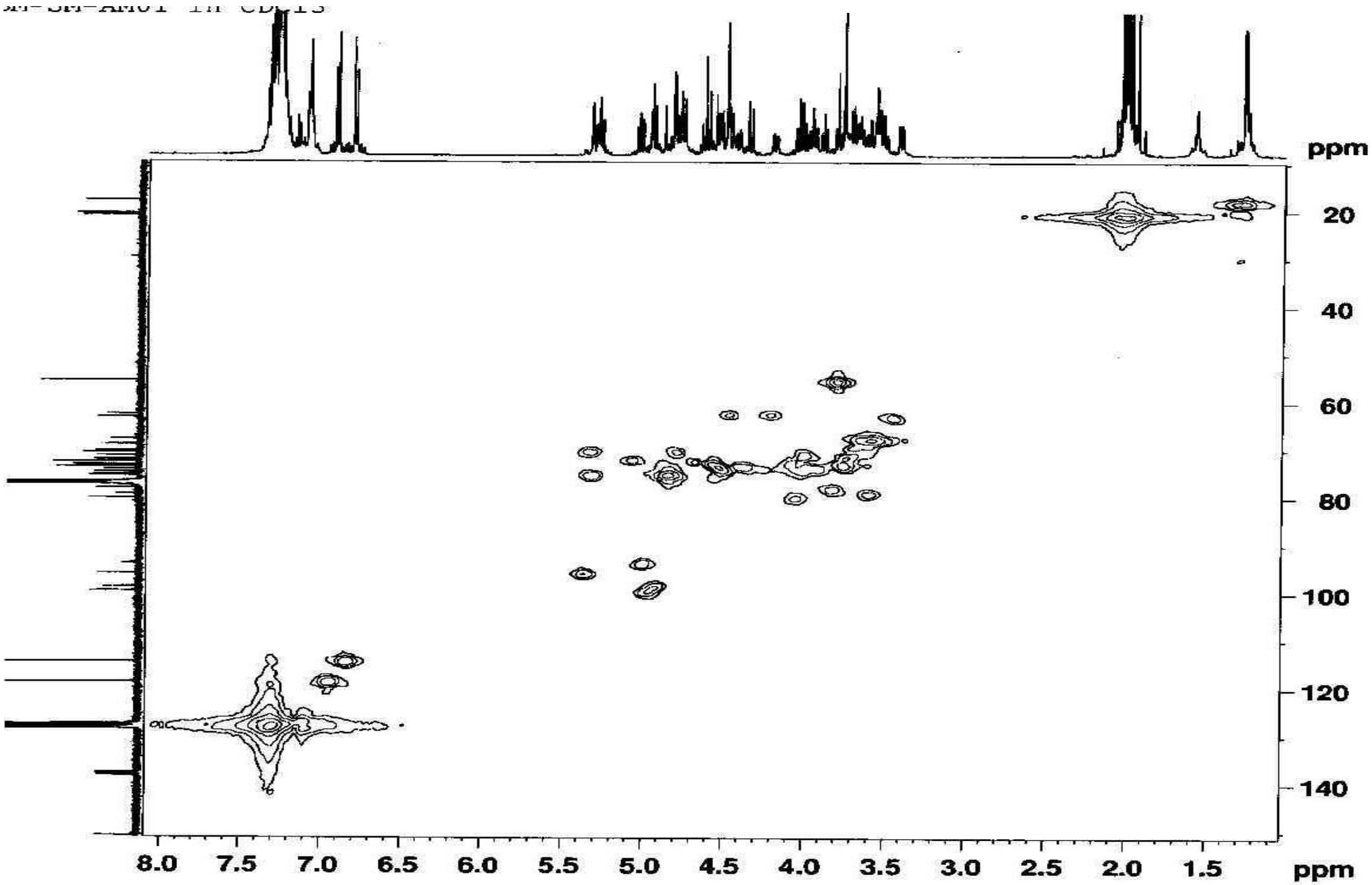

2D HMQC NMR spectrum of 4-methoxyphenyl (2-azido-3,4,6-tri-*O*-benzyl-2-deoxy-α-D-glucopyranosyl)-(1→3)-(2,4-di-*O*-benzyl-α-L-rhamnopyranosyl)-(1→4)-(2,3-di-*O*-acetyl-6-*O*-benzyl-α-D-glucopyranosyl)-(1→4)-2,3,6-tri-*O*-acetyl-β-D-glucopyranoside (**8**).

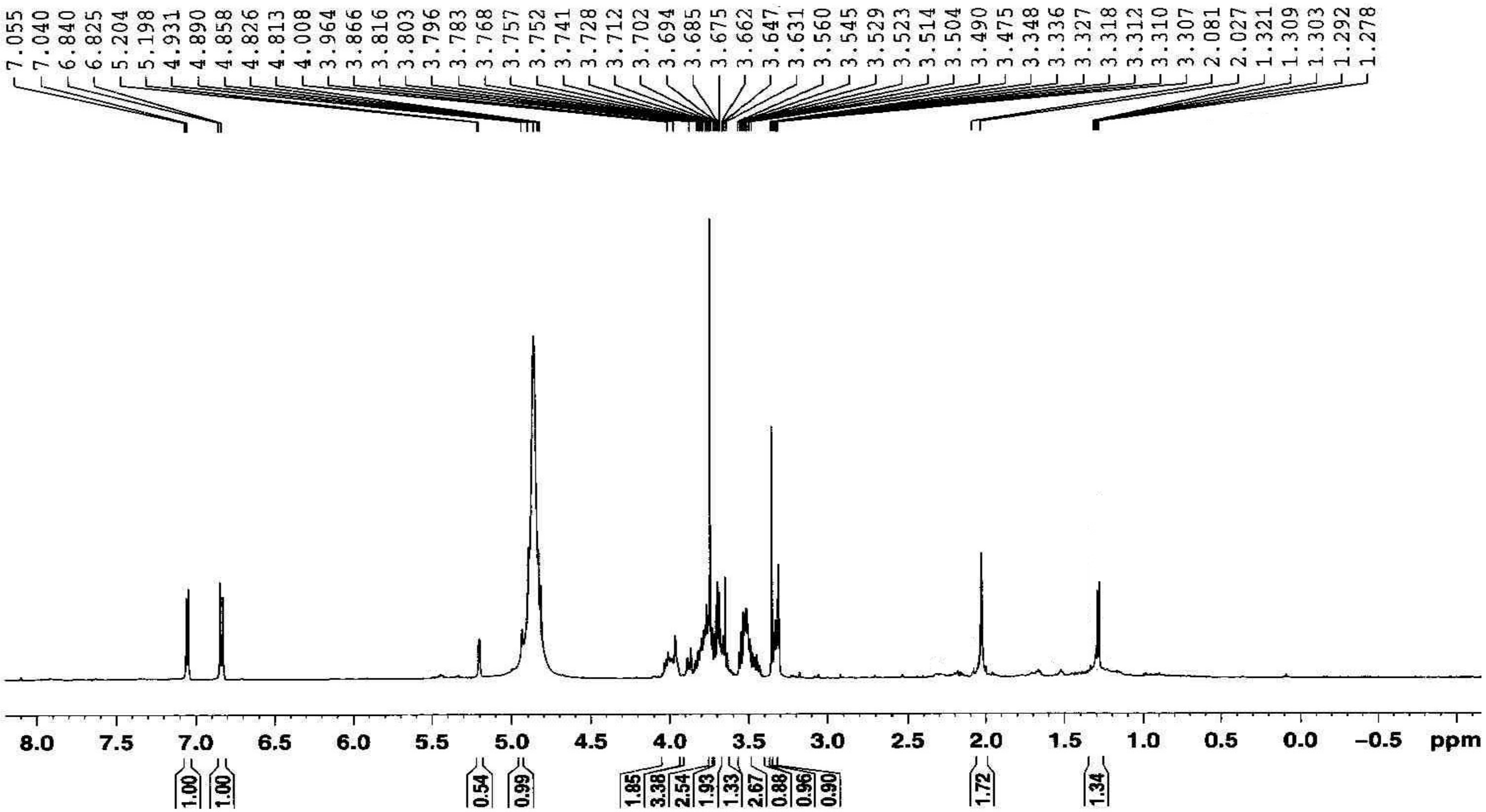

^{1}H NMR spectrum of 4-methoxyphenyl (2-acetamido-2-deoxy-α-D-glucopyranosyl)-(1→3)-(α-L-rhamnopyranosyl)-(1→4)-(α-D-glucopyranosyl)-(1→4)-sodium β-D-glucopyranosiduronate (**1**) (CD_3OD).

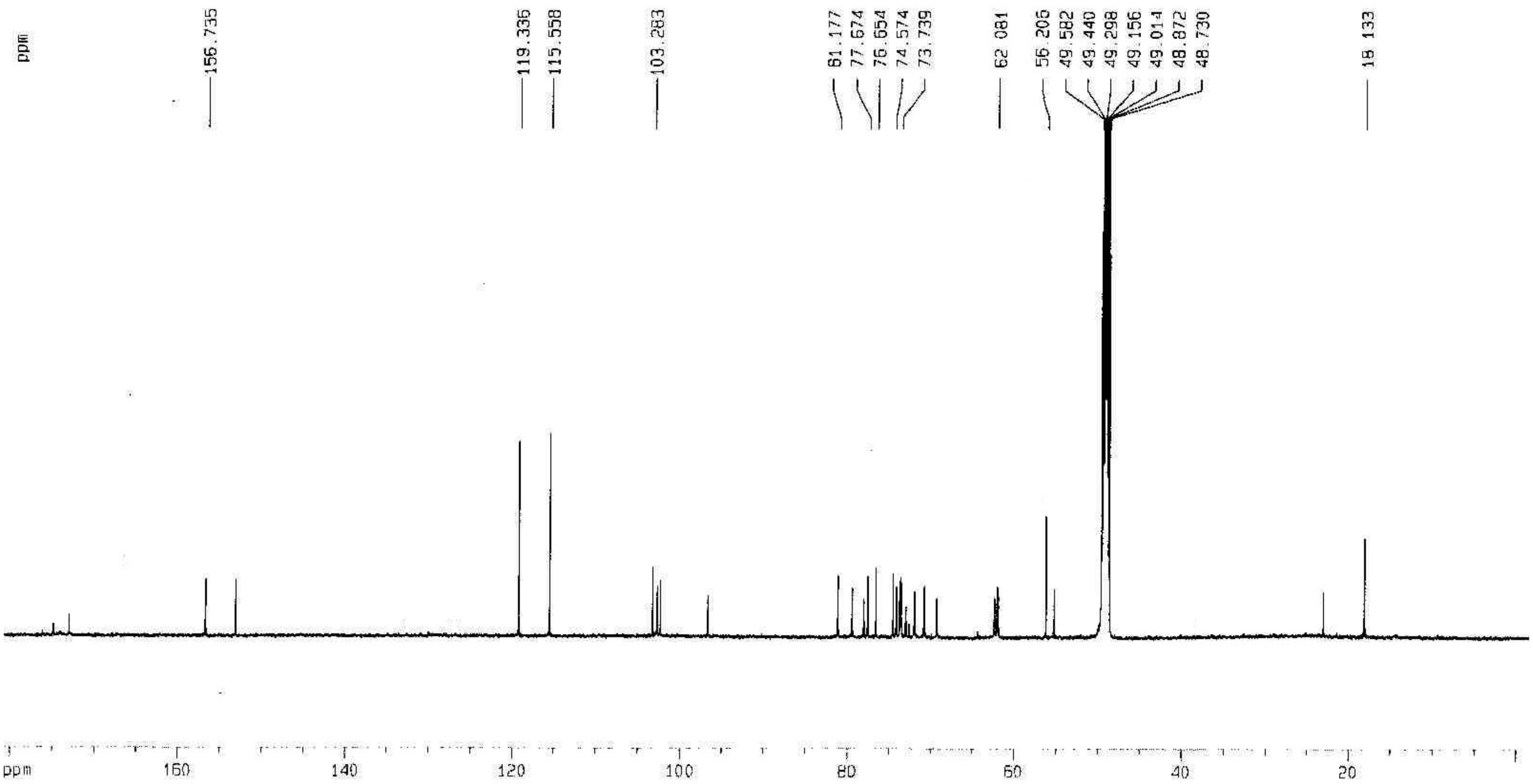

^{13}C NMR spectrum of 4-methoxyphenyl (2-acetamido-2-deoxy-α-D-glucopyranosyl)-(1→3)-(α-L-rhamnopyranosyl)-(1→4)-(α-D-glucopyranosyl)-(1→4)-sodium β-D-glucopyranosiduronate (**1**) (CD_3OD).

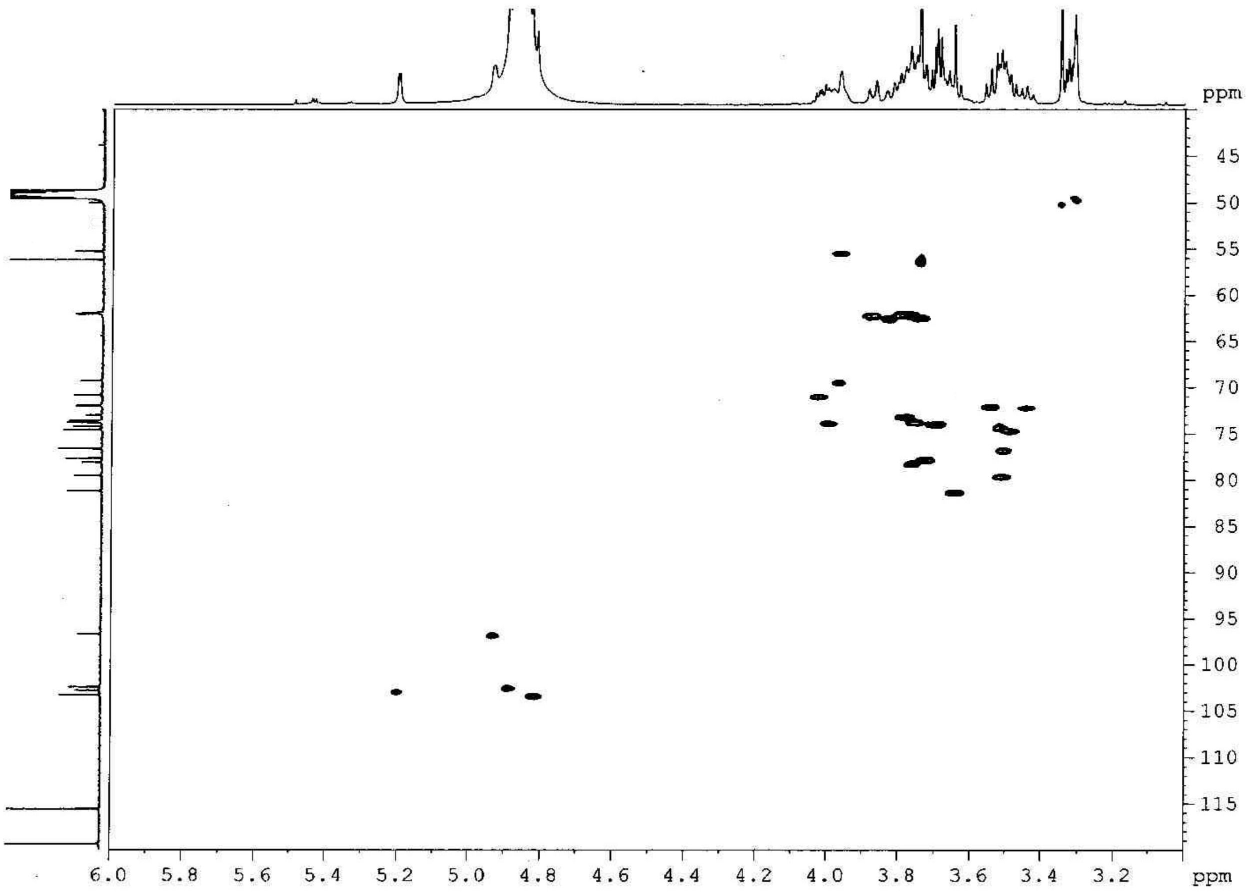

2D HMQC NMR spectrum of 4-methoxyphenyl (2-acetamido-2-deoxy-α-D-glucopyranosyl)-(1→3)-(α-L-rhamnopyranosyl)-(1→4)-(α-D-glucopyranosyl)-(1→4)-sodium β-D-glucopyranosiduronate (**1**) (CD_3OD) (expanded region).
